# CYP3A7*1C allele: linking premenopausal oestrone and progesterone levels with risk of hormone receptor-positive breast cancers

**DOI:** 10.1038/s41416-020-01185-w

**Published:** 2021-01-26

**Authors:** Nichola Johnson, Sarah Maguire, Anna Morra, Pooja Middha Kapoor, Katarzyna Tomczyk, Michael E. Jones, Minouk J. Schoemaker, Clare Gilham, Manjeet K. Bolla, Qin Wang, Joe Dennis, Thomas U. Ahearn, Irene L. Andrulis, Hoda Anton-Culver, Natalia N. Antonenkova, Volker Arndt, Kristan J. Aronson, Annelie Augustinsson, Caroline Baynes, Laura E. Beane Freeman, Matthias W. Beckmann, Javier Benitez, Marina Bermisheva, Carl Blomqvist, Bram Boeckx, Natalia V. Bogdanova, Stig E. Bojesen, Hiltrud Brauch, Hermann Brenner, Barbara Burwinkel, Daniele Campa, Federico Canzian, Jose E. Castelao, Stephen J. Chanock, Georgia Chenevix-Trench, Christine L. Clarke, NBCS Collaborators, Anne-Lise Børresen-Dale, Grethe I. Grenaker Alnæs, Kristine K. Sahlberg, Lars Ottestad, Rolf Kåresen, Ellen Schlichting, Marit Muri Holmen, Toril Sauer, Vilde Haakensen, Olav Engebråten, Bjørn Naume, Alexander Fosså, Cecile E. Kiserud, Kristin V. Reinertsen, Åslaug Helland, Margit Riis, Jürgen Geisler, Don M. Conroy, Fergus J. Couch, Angela Cox, Simon S. Cross, Kamila Czene, Thilo Dörk, A. Heather Eliassen, Christoph Engel, D. Gareth Evans, Peter A. Fasching, Jonine Figueroa, Giuseppe Floris, Henrik Flyger, Manuela Gago-Dominguez, Susan M. Gapstur, Montserrat García-Closas, Mia M. Gaudet, Graham G. Giles, Mark S. Goldberg, Anna González-Neira, AOCS Group, David D. L. Bowtell, Anna deFazio, Penelope M. Webb, Georgia Chenevix-Trench, Pascal Guénel, Eric Hahnen, Christopher A. Haiman, Niclas Håkansson, Per Hall, Ute Hamann, Patricia A. Harrington, Steven N. Hart, Maartje J. Hooning, John L. Hopper, Anthony Howell, David J. Hunter, ABCTB Investigators, Christine Clarke, Deborah Marsh, Rodney Scott, Robert Baxter, Desmond Yip, Jane Carpenter, Alison Davis, Nirmala Pathmanathan, Peter Simpson, Dinny Graham, Mythily Sachchithananthan, kConFab Investigators, David Amor, Lesley Andrews, Yoland Antill, Rosemary Balleine, Jonathan Beesley, Ian Bennett, Michael Bogwitz, Leon Botes, Meagan Brennan, Melissa Brown, Michael Buckley, Jo Burke, Phyllis Butow, Liz Caldon, Ian Campbell, Deepa Chauhan, Manisha Chauhan, Georgia Chenevix-Trench, Alice Christian, Paul Cohen, Alison Colley, Ashley Crook, James Cui, Margaret Cummings, Sarah-Jane Dawson, Anna DeFazio, Martin Delatycki, Rebecca Dickson, Joanne Dixon, Ted Edkins, Stacey Edwards, Gelareh Farshid, Andrew Fellows, Georgina Fenton, Michael Field, James Flanagan, Peter Fong, Laura Forrest, Stephen Fox, Juliet French, Michael Friedlander, Clara Gaff, Mike Gattas, Peter George, Sian Greening, Marion Harris, Stewart Hart, Nick Hayward, John Hopper, Cass Hoskins, Clare Hunt, Paul James, Mark Jenkins, Alexa Kidd, Judy Kirk, Jessica Koehler, James Kollias, Sunil Lakhani, Mitchell Lawrence, Geoff Lindeman, Lara Lipton, Liz Lobb, Graham Mann, Deborah Marsh, Sue Anne McLachlan, Bettina Meiser, Roger Milne, Sophie Nightingale, Shona O’Connell, Sarah O’Sullivan, David Gallego Ortega, Nick Pachter, Briony Patterson, Amy Pearn, Kelly Phillips, Ellen Pieper, Edwina Rickard, Bridget Robinson, Mona Saleh, Elizabeth Salisbury, Christobel Saunders, Jodi Saunus, Rodney Scott, Clare Scott, Adrienne Sexton, Andrew Shelling, Peter Simpson, Melissa Southey, Amanda Spurdle, Jessica Taylor, Renea Taylor, Heather Thorne, Alison Trainer, Kathy Tucker, Jane Visvader, Logan Walker, Rachael Williams, Ingrid Winship, Mary Ann Young, Agnes Jager, Anna Jakubowska, Esther M. John, Rudolf Kaaks, Renske Keeman, Elza Khusnutdinova, Cari M. Kitahara, Veli-Matti Kosma, Stella Koutros, Peter Kraft, Vessela N. Kristensen, Allison W. Kurian, Diether Lambrechts, Loic Le Marchand, Martha Linet, Jan Lubiński, Arto Mannermaa, Siranoush Manoukian, Sara Margolin, John W. M. Martens, Dimitrios Mavroudis, Rebecca Mayes, Alfons Meindl, Roger L. Milne, Susan L. Neuhausen, Heli Nevanlinna, William G. Newman, Sune F. Nielsen, Børge G. Nordestgaard, Nadia Obi, Andrew F. Olshan, Janet E. Olson, Håkan Olsson, Ester Orban, Tjoung-Won Park-Simon, Paolo Peterlongo, Dijana Plaseska-Karanfilska, Katri Pylkäs, Gad Rennert, Hedy S. Rennert, Kathryn J. Ruddy, Emmanouil Saloustros, Dale P. Sandler, Elinor J. Sawyer, Rita K. Schmutzler, Christopher Scott, Xiao-Ou Shu, Jacques Simard, Snezhana Smichkoska, Christof Sohn, Melissa C. Southey, John J. Spinelli, Jennifer Stone, Rulla M. Tamimi, Jack A. Taylor, Rob A. E. M. Tollenaar, Ian Tomlinson, Melissa A. Troester, Thérèse Truong, Celine M. Vachon, Elke M. van Veen, Sophia S. Wang, Clarice R. Weinberg, Camilla Wendt, Hans Wildiers, Robert Winqvist, Alicja Wolk, Wei Zheng, Argyrios Ziogas, Alison M. Dunning, Paul D. P. Pharoah, Douglas F. Easton, A. Forbes Howie, Julian Peto, Isabel dos-Santos-Silva, Anthony J. Swerdlow, Jenny Chang-Claude, Marjanka K. Schmidt, Nick Orr, Olivia Fletcher

**Affiliations:** 1 The Breast Cancer Now Toby Robins Research Centre, The Institute of Cancer Research, London, UK; 2 Centre for Cancer Research and Cell Biology, Queen’s University Belfast, Belfast, Ireland UK; 3 Division of Molecular Pathology, The Netherlands Cancer Institute - Antoni van Leeuwenhoek Hospital, Amsterdam, The Netherlands; 4 Division of Cancer Epidemiology, German Cancer Research Center (DKFZ), Heidelberg, Germany; 5 Faculty of Medicine, University of Heidelberg, Heidelberg, Germany; 6 Division of Genetics and Epidemiology, The Institute of Cancer Research, London, UK; 7 Department of Non-Communicable Disease Epidemiology, London School of Hygiene and Tropical Medicine, London, UK; 8 Centre for Cancer Genetic Epidemiology, Department of Public Health and Primary Care, University of Cambridge, Cambridge, UK; 9 Division of Cancer Epidemiology and Genetics, National Cancer Institute, National Institutes of Health, Department of Health and Human Services, Bethesda, MD USA; 10 Fred A. Litwin Center for Cancer Genetics, Lunenfeld-Tanenbaum Research Institute of Mount Sinai Hospital, Toronto, ON Canada; 11 Department of Molecular Genetics, University of Toronto, Toronto, ON Canada; 12 Department of Epidemiology, Genetic Epidemiology Research Institute, University of California Irvine, Irvine, CA USA; 13 N.N. Alexandrov Research Institute of Oncology and Medical Radiology, Minsk, Belarus; 14 Division of Clinical Epidemiology and Aging Research, German Cancer Research Center (DKFZ), Heidelberg, Germany; 15 Department of Public Health Sciences, and Cancer Research Institute, Queen’s University, Kingston, ON Canada; 16 Department of Cancer Epidemiology, Clinical Sciences, Lund University, Lund, Sweden; 17 Centre for Cancer Genetic Epidemiology, Department of Oncology, University of Cambridge, Cambridge, UK; 18 Department of Gynecology and Obstetrics, Comprehensive Cancer Center ER-EMN, University Hospital Erlangen, Friedrich-Alexander-University Erlangen-Nuremberg, Erlangen, Germany; 19 Centro de Investigación en Red de Enfermedades Raras (CIBERER), Madrid, Spain; 20 Human Cancer Genetics Programme, Spanish National Cancer Research Centre (CNIO), Madrid, Spain; 21 Institute of Biochemistry and Genetics, Ufa Federal Research Centre of the Russian Academy of Sciences, Ufa, Russia; 22 Department of Oncology, Helsinki University Hospital, University of Helsinki, Helsinki, Finland; 23 Department of Oncology, Örebro University Hospital, Örebro, Sweden; 24 VIB Center for Cancer Biology, Leuven, Belgium; 25 Laboratory for Translational Genetics, Department of Human Genetics, University of Leuven, Leuven, Belgium; 26 Department of Radiation Oncology, Hannover Medical School, Hannover, Germany; 27 Gynaecology Research Unit, Hannover Medical School, Hannover, Germany; 28 Copenhagen General Population Study, Herlev and Gentofte Hospital, Copenhagen University Hospital, Herlev, Denmark; 29 Department of Clinical Biochemistry, Herlev and Gentofte Hospital, Copenhagen University Hospital, Herlev, Denmark; 30 Faculty of Health and Medical Sciences, University of Copenhagen, Copenhagen, Denmark; 31 Dr. Margarete Fischer-Bosch-Institute of Clinical Pharmacology, Stuttgart, Germany; 32 iFIT-Cluster of Excellence, University of Tübingen, Tübingen, Germany; 33 German Cancer Consortium (DKTK), German Cancer Research Center (DKFZ), Heidelberg, Germany; 34 Division of Preventive Oncology, German Cancer Research Center (DKFZ) and National Center for Tumor Diseases (NCT), Heidelberg, Germany; 35 Molecular Epidemiology Group, C080, German Cancer Research Center (DKFZ), Heidelberg, Germany; 36 Molecular Biology of Breast Cancer, University Womens Clinic Heidelberg, University of Heidelberg, Heidelberg, Germany; 37 Department of Biology, University of Pisa, Pisa, Italy; 38 Genomic Epidemiology Group, German Cancer Research Center (DKFZ), Heidelberg, Germany; 39 Oncology and Genetics Unit, Instituto de Investigacion Sanitaria Galicia Sur (IISGS), Xerencia de Xestion Integrada de Vigo-SERGAS, Vigo, Spain; 40 Department of Genetics and Computational Biology, QIMR Berghofer Medical Research Institute, Brisbane, Queensland Australia; 41 Westmead Institute for Medical Research, University of Sydney, Sydney, New South Wales Australia; 42 Department of Laboratory Medicine and Pathology, Mayo Clinic, Rochester, MN USA; 43 Sheffield Institute for Nucleic Acids (SInFoNiA), Department of Oncology and Metabolism, University of Sheffield, Sheffield, UK; 44 Academic Unit of Pathology, Department of Neuroscience, University of Sheffield, Sheffield, UK; 45 Department of Medical Epidemiology and Biostatistics, Karolinska Institutet, Stockholm, Sweden; 46 Channing Division of Network Medicine, Department of Medicine, Brigham and Women’s Hospital and Harvard Medical School, Boston, MA USA; 47 Department of Epidemiology, Harvard T.H. Chan School of Public Health, Boston, MA USA; 48 Institute for Medical Informatics, Statistics and Epidemiology, University of Leipzig, Leipzig, Germany; 49 LIFE - Leipzig Research Centre for Civilization Diseases, University of Leipzig, Leipzig, Germany; 50 Division of Evolution and Genomic Sciences, School of Biological Sciences, Faculty of Biology, Medicine and Health, University of Manchester, Manchester Academic Health Science Centre, Manchester, UK; 51 North West Genomics Laboratory Hub, Manchester Centre for Genomic Medicine, St Mary’s Hospital, Manchester University NHS Foundation Trust, Manchester Academic Health Science Centre, Manchester, UK; 52 David Geffen School of Medicine, Department of Medicine Division of Hematology and Oncology, University of California at Los Angeles, Los Angeles, CA USA; 53 Usher Institute of Population Health Sciences and Informatics, The University of Edinburgh, Edinburgh, UK; 54 Cancer Research UK Edinburgh Centre, The University of Edinburgh, Edinburgh, UK; 55 Leuven Multidisciplinary Breast Center, Department of Oncology, Leuven Cancer Institute, University Hospitals Leuven, Leuven, Belgium; 56 Department of Breast Surgery, Herlev and Gentofte Hospital, Copenhagen University Hospital, Herlev, Denmark; 57 Fundación Pública Galega de Medicina Xenómica, Instituto de Investigación Sanitaria de Santiago de Compostela (IDIS), Complejo Hospitalario Universitario de Santiago, SERGAS, Santiago de Compostela, Spain; 58 Moores Cancer Center, University of California San Diego, La Jolla, CA USA; 59 Behavioral and Epidemiology Research Group, American Cancer Society, Atlanta, GA USA; 60 Cancer Epidemiology Division, Cancer Council Victoria, Melbourne, Victoria Australia; 61 Centre for Epidemiology and Biostatistics, Melbourne School of Population and Global Health, The University of Melbourne, Melbourne, Victoria Australia; 62 Precision Medicine, School of Clinical Sciences at Monash Health, Monash University, Clayton, Victoria Australia; 63 Department of Medicine, McGill University, Montréal, QC Canada; 64 Division of Clinical Epidemiology, Royal Victoria Hospital, McGill University, Montréal, QC Canada; 65 Center for Research in Epidemiology and Population Health (CESP), Team Exposome and Heredity, INSERM, University Paris-Saclay, Villejuif, France; 66 Center for Familial Breast and Ovarian Cancer, Faculty of Medicine and University Hospital Cologne, University of Cologne, Cologne, Germany; 67 Center for Integrated Oncology (CIO), Faculty of Medicine and University Hospital Cologne, University of Cologne, Cologne, Germany; 68 Department of Preventive Medicine, Keck School of Medicine, University of Southern California, Los Angeles, CA USA; 69 Institute of Environmental Medicine, Karolinska Institutet, Stockholm, Sweden; 70 Department of Oncology, Södersjukhuset, Stockholm, Sweden; 71 Molecular Genetics of Breast Cancer, German Cancer Research Center (DKFZ), Heidelberg, Germany; 72 Department of Health Sciences Research, Mayo Clinic, Rochester, MN USA; 73 Department of Medical Oncology, Erasmus MC Cancer Institute, Rotterdam, The Netherlands; 74 Division of Cancer Sciences, University of Manchester, Manchester, UK; 75 Nuffield Department of Population Health, University of Oxford, Oxford, UK; 76 Department of Genetics and Pathology, Pomeranian Medical University, Szczecin, Poland; 77 Independent Laboratory of Molecular Biology and Genetic Diagnostics, Pomeranian Medical University, Szczecin, Poland; 78 Department of Epidemiology & Population Health, Stanford University School of Medicine, Stanford, CA USA; 79 Department of Medicine, Division of Oncology, Stanford Cancer Institute, Stanford University School of Medicine, Stanford, CA USA; 80 Department of Genetics and Fundamental Medicine, Bashkir State University, Ufa, Russia; 81 Radiation Epidemiology Branch, Division of Cancer Epidemiology and Genetics, National Cancer Institute, Bethesda, MD USA; 82 Translational Cancer Research Area, University of Eastern Finland, Kuopio, Finland; 83 Institute of Clinical Medicine, Pathology and Forensic Medicine, University of Eastern Finland, Kuopio, Finland; 84 Biobank of Eastern Finland, Kuopio University Hospital, Kuopio, Finland; 85 Program in Genetic Epidemiology and Statistical Genetics, Harvard T.H. Chan School of Public Health, Boston, MA USA; 86 Institute of Clinical Medicine, Faculty of Medicine, University of Oslo, Oslo, Norway; 87 Department of Medical Genetics, Oslo University Hospital and University of Oslo, Oslo, Norway; 88 Epidemiology Program, University of Hawaii Cancer Center, Honolulu, HI USA; 89 Unit of Medical Genetics, Department of Medical Oncology and Hematology, Fondazione IRCCS Istituto Nazionale dei Tumori di Milano, Milan, Italy; 90 Department of Clinical Science and Education, Södersjukhuset, Karolinska Institutet, Stockholm, Sweden; 91 Department of Medical Oncology, University Hospital of Heraklion, Heraklion, Greece; 92 Department of Gynecology and Obstetrics, University of Munich, Campus Großhadern, Munich, Germany; 93 Department of Population Sciences, Beckman Research Institute of City of Hope, Duarte, CA USA; 94 Department of Obstetrics and Gynecology, Helsinki University Hospital, University of Helsinki, Helsinki, Finland; 95 Institute of Medical Biometry and Epidemiology, University Medical Center Hamburg-Eppendorf, Hamburg, Germany; 96 Department of Epidemiology, Gillings School of Global Public Health and UNC Lineberger Comprehensive Cancer Center, University of North Carolina at Chapel Hill, Chapel Hill, NC USA; 97 Cancer Epidemiology Group, University Cancer Center Hamburg (UCCH), University Medical Center Hamburg-Eppendorf, Hamburg, Germany; 98 Genome Diagnostics Program, IFOM - the FIRC Institute of Molecular Oncology, Milan, Italy; 99 Research Centre for Genetic Engineering and Biotechnology ‘Georgi D. Efremov’, MASA, Skopje, Republic of North Macedonia; 100 Laboratory of Cancer Genetics and Tumor Biology, Cancer and Translational Medicine Research Unit, Biocenter Oulu, University of Oulu, Oulu, Finland; 101 Laboratory of Cancer Genetics and Tumor Biology, Northern Finland Laboratory Centre Oulu, Oulu, Finland; 102 Clalit National Cancer Control Center, Carmel Medical Center and Technion Faculty of Medicine, Haifa, Israel; 103 Department of Oncology, Mayo Clinic, Rochester, MN USA; 104 Department of Oncology, University Hospital of Larissa, Larissa, Greece; 105 Epidemiology Branch, National Institute of Environmental Health Sciences, NIH, Research Triangle Park, NC USA; 106 School of Cancer & Pharmaceutical Sciences, Comprehensive Cancer Centre, Guy’s Campus, King’s College London, London, UK; 107 Center for Molecular Medicine Cologne (CMMC), Faculty of Medicine and University Hospital Cologne, University of Cologne, Cologne, Germany; 108 Division of Epidemiology, Department of Medicine, Vanderbilt Epidemiology Center, Vanderbilt-Ingram Cancer Center, Vanderbilt University School of Medicine, Nashville, TN USA; 109 Genomics Center, Centre Hospitalier Universitaire de Québec - Université Laval Research Center, Québec City, QC Canada; 110 Ss. Cyril and Methodius University in Skopje, Medical Faculty, University Clinic of Radiotherapy and Oncology, Skopje, Republic of North Macedonia; 111 National Center for Tumor Diseases, University Hospital and German Cancer Research Center, Heidelberg, Germany; 112 Department of Clinical Pathology, The University of Melbourne, Melbourne, Victoria Australia; 113 Population Oncology, BC Cancer, Vancouver, BC Canada; 114 School of Population and Public Health, University of British Columbia, Vancouver, BC Canada; 115 The Curtin UWA Centre for Genetic Origins of Health and Disease, Curtin University and University of Western Australia, Perth, Western Australia Australia; 116 Department of Population Health Sciences, Weill Cornell Medicine, New York, NY USA; 117 Epigenetic and Stem Cell Biology Laboratory, National Institute of Environmental Health Sciences, NIH, Research Triangle Park, NC USA; 118 Department of Surgery, Leiden University Medical Center, Leiden, The Netherlands; 119 Institute of Cancer and Genomic Sciences, University of Birmingham, Birmingham, UK; 120 Wellcome Trust Centre for Human Genetics and Oxford NIHR Biomedical Research Centre, University of Oxford, Oxford, UK; 121 Department of Health Science Research, Division of Epidemiology, Mayo Clinic, Rochester, MN USA; 122 Department of Computational and Quantitative Medicine, City of Hope, Duarte, CA USA; 123 City of Hope Comprehensive Cancer Center, City of Hope, Duarte, CA USA; 124 Biostatistics and Computational Biology Branch, National Institute of Environmental Health Sciences, NIH, Research Triangle Park, NC USA; 125 Department of Surgical Sciences, Uppsala University, Uppsala, Sweden; 126 MRC Centre for Reproductive Health, University of Edinburgh, Edinburgh, UK; 127 Division of Breast Cancer Research, The Institute of Cancer Research, London, UK; 128 Division of Psychosocial Research and Epidemiology, The Netherlands Cancer Institute - Antoni van Leeuwenhoek hospital, Amsterdam, The Netherlands; 129 Department of Cancer Genetics, Institute for Cancer Research, Oslo University Hospital-Radiumhospitalet, Oslo, Norway; 130 Department of Research, Vestre Viken Hospital, Drammen, Norway; 131 Section for Breast- and Endocrine Surgery, Department of Cancer, Division of Surgery, Cancer and Transplantation Medicine, Oslo University Hospital-Ullevål, Oslo, Norway; 132 Department of Radiology and Nuclear Medicine, Oslo University Hospital, Oslo, Norway; 133 Department of Pathology at Akershus University hospital, Lørenskog, Norway; 134 Department of Oncology, Division of Surgery and Cancer and Transplantation Medicine, Oslo University Hospital-Radiumhospitalet, Oslo, Norway; 135 Department of Tumor Biology, Institute for Cancer Research, Oslo University Hospital, Oslo, Norway; 136 National Advisory Unit on Late Effects after Cancer Treatment, Department of Oncology, Oslo University Hospital, Oslo, Norway; 137 Department of Oncology, Akershus University Hospital, Lørenskog, Norway; 138 Breast Cancer Research Consortium, Oslo University Hospital, Oslo, Norway; 139 Peter MacCallum Cancer Centre, Melbourne, 3000 VIC Australia; 140 Sir Peter MacCallum Department of Oncology, The University of Melbourne, Parkville, 3010 VIC Australia; 141 Kinghorn Cancer Centre, Garvan Institute for Medical Research, Darlinghurst, 2010 NSW Australia; 142 Centre for Cancer Research, The Westmead Institute for Medical Research, Westmead, 2145 NSW Australia; 143 Department of Gynaecological Oncology, Westmead Hospital, Westmead, 2145 NSW Australia; 144 The University of Sydney, Sydney, 2052 NSW Australia; 145 QIMR Berghofer Medical Research Institute, Locked Bag 2000 Royal Brisbane Hospital, Brisbane, Queensland 4029 Australia; 146 Centre for Cancer Research, The Westmead Institute for Medical Research, The University of Sydney, Sydney, NSW Australia; 147 University of Technology Sydney, Translational Oncology Group, School of Life Sciences, Faculty of Science, Ultimo, NSW Australia; 148 School of Biomedical Sciences, University of Newcastle, Newcastle, Australia; 149 Hunter Medical Research Institute and NSW Health Pathology North, Newcastle, Australia; 150 Kolling Institute of Medical Research, University of Sydney, St Leonards, NSW Australia; 151 Epigenetics & Transcription Laboratory Melanie Swan Memorial Translational Centre, Sci-Tech, University of Canberra, Canberra, ACT Australia; 152 Department of Medical Oncology, The Canberra Hospital, Garran, ACT Australia; 153 Scientific Platforms, The Westmead Institute for Medical Research, The University of Sydney, Sydney, NSW Australia; 154 The Canberra Hospital, Garran, ACT Australia; 155 The Australian National University, Canberra, ACT Australia; 156 Westmead Breast Cancer Institute, Western Sydney Local Health District, Westmead, New South Wales Australia; 157 University of Sydney, Western Clinical School, Westmead, New South Wales Australia; 158 UQ Centre for Clinical Research, Faculty of Medicine, The University of Queensland, Herston, QLD Australia; 159 Genetic Health Services, Victoria Royal Children’s Hospital, Melbourne, VIC 3050 Australia; 160 Hereditary Cancer Clinic, Prince of Wales Hospital, Randwick, NSW 2031 Australia; 161 Dept. Haem and Medical Oncology, Peter MacCallum Cancer Centre, St Andrews Place, East Melbourne, VIC 3002 Australia; 162 Department of Translational Oncology, c/o Department of Medical Oncology, Westmead Hospital, Westmead, NSW 2145 Australia; 163 Queensland Institute of Medical Research, Herston Road, Herston, Qld 4002 Australia; 164 Silverton Place, 101 Wickham Terrace, Brisbane, QLD 4000 Australia; 165 Familial Cancer Centre, The Royal Melbourne Hospital, Grattan Street, Parkville, Victoria 3050 Australia; 166 Hereditary Cancer Centre, Prince of Wales Hospital, Barker St, Randwick, NSW 2031 Australia; 167 NSW Breast Cancer Institute, PO Box 143, Westmead, NSW 2145 Australia; 168 Department of Biochemistry, University of Queensland, St. Lucia, QLD 4072 Australia; 169 Molecular and Cytogenetics Unit, Prince of Wales Hospital, Randwick, NSW 2031 Australia; 170 Royal Hobart Hospital, GPO Box 1061L, Hobart, TAS 7001 Australia; 171 Medical Psychology Unit, Royal Prince Alfred Hospital, Camperdown, NSW 2204 Australia; 172 Replication and Genome Stability, Cancer Division, Garvan Institute of Medical Research, 370 Victoria Street, Darlinghurst, NSW 2010 Australia; 173 Peter MacCallum Cancer Centre St Andrew’s Place, East Melbourne, VIC 3002 Australia; 174 School of Psychology, Brennan McCallum (Building A18), University of Sydney, Sydney, NSW 2006 Australia; 175 St Vincents Hospital, Cancer Genetics Clinic, The Kinghorn Cancer Centre, Sydney, NSW 2010 Australia; 176 Queensland Institute of Medical Research, Royal Brisbane Hospital, Herston, QLD 4029 Australia; 177 Genetics Department, Central Region Genetics Service, Wellington Hospital, Wellington, New Zealand; 178 Gynaecological Cancer Research, St John of God Subiaco Hospital, 12 Salvado Road, Subiaco, WA 6008 Australia; 179 Department of Clinical Genetics, Liverpool Health Service, PO Box 103, Liverpool, NSW 2170 Australia; 180 Department of Clinical Genetics, Level 3E, Royal North Shore Hospital, St Leonards, NSW 2065 Australia; 181 Epidemiology and Preventive Medicine, Monash University, Prahan, Vic 3004 Australia; 182 Department of Pathology, University of Queensland Medical School, Herston, NSW 4006 Australia; 183 Molecular Genetics Department, Cambridge University, Cambridge, England; 184 Dept. Gynaecological Oncology, Westmead Institute for Cancer Research, Westmead Hospital, Westmead, NSW 2145 Australia; 185 Clinical Genetics, Austin Health, Heidelberg Repatriation Hospital, PO Box 5444, Heidelberg West, Vic 3081 Australia; 186 Level 2, Block 51, Royal North Shore Hospital, North Shore, NSW 2408 Australia; 187 Central Regional Genetic Services, Wellington Hospital, Private Bag, 7902 Wellington, New Zealand; 188 Clinical Chemistry, Princess Margret Hospital for Children, Box D184, Perth, WA 6001 Australia; 189 Department of Biochemistry and Molecular Biology, University of Queensland, St Lucia, Qld 4072 Australia; 190 Tissue Pathology, IMVS, Adelaide, SA 5000 Australia; 191 Molecular Diagnostic Development, Pathology Department, Peter MacCallum Cancer Centre, Melbourne East, Melbourne, Vic 3002 Australia; 192 South West Family Cancer Clinic, Liverpool Hospital, Liverpool BC, NSW 1871 Australia; 193 Royal North Shore Hospital, Level 2, Vindin House, St Leonards, NSW 2065 Australia; 194 Epigenetics Unit, Department of Surgery and Oncology, Imperial College London, London, W12 0NN UK; 195 Medical Oncology Department, Regional Cancer and Blood Services, Level 1 Building 7, Auckland City Hospital, 2 Park Rd., Grafton, Auckland, 1023 New Zealand; 196 Psychosocial Cancer Genetics Research Group, Parkville Familial Cancer Centre, 305 Grattan Street, Melbourne, Vic 3000 Australia; 197 Pathology Department, Level 1, Peter MacCallum Cancer Centre, St Andrew’s Place, East Melbourne, Vic 3002 Australia; 198 School of Molecular and Microbial Sciences, University of Queensland, St Lucia, Qld 4072 Australia; 199 Department of Medical Oncology, Prince of Wales Hospital, Randwick, NSW 2031 Australia; 200 Victorian Clinical Genetics Service, Royal Melbourne Hospital, Parkville, VIC 3052 Australia; 201 Queensland Clinical Genetic Service, Royal Children’s Hospital, Bramston Terrace, Herston, QLD 4020 Australia; 202 Clinical Biochemistry Unit, Canterbury Health Labs, PO Box 151, Christchurch, New Zealand; 203 Illawarra Cancer Centre, Wollongong Hospital, Private Mail Bag 8808, South Coast Mail Centre, Wollongong, NSW 2521 Australia; 204 Familial Cancer Clinic, Peter MacCallum Cancer Centre, St Andrews Place, East Melbourne, VIC 3002 Australia; 205 Breast and Ovarian Cancer Genetics, Monash Medical Centre, 871 Centre Road, Bentleigh East, VIC 3165 Australia; 206 Queensland Institute for Medical Research, Royal Brisbane Hospital, Post Office, Herston, QLD 4029 Australia; 207 Centre for M.E.G.A. Epidemiology, University of Melbourne, Level 1, 723 Swanston Street, Carlton, VIC 3010 Australia; 208 Parkville Familial Cancer Centre, Peter MacCallum Cancer Centre & The Royal Melbourne Hospital, Melbourne, VIC 3000 Australia; 209 Southern Health Familial Cancer Centre, Monash Medical Centre, Special Medicine Building, 246 Clayton Rd, Clayton, Victoria 3168 Australia; 210 Genetic Health Sevices, Monash Medical Centre, Clayton, Vic 3168 Australia; 211 Centre for M.E.G.A. Epidemiology, The University of Melbourne, 723 Swanston Street, Carlton, VIC 3053 Australia; 212 Clinical Genetics Departments, Central Regional Genetics Service, Wellington Hospital, Wellington, New Zealand; 213 Familial Cancer Service, Department of Medicine, Westmead Hospital, Westmead, NSW 2145 Australia; 214 Breast Endocrine and Surgical Unit, Royal Adelaide Hospital, North Terrace, SA 5000 Australia; 215 UQ Centre for Clinical Research, Level 6, Building 71/918, University of Queensland, The Royal Brisbane & Women’s Hospital, Herston, QLD 4029 Australia; 216 Prostate Cancer Research Program, 19 Innovation Walk, Level 3, Monash University, Clayton, VIC 3800 Australia; 217 Breast Cancer Laboratory, Walter and Eliza Hall Institute, PO Royal Melbourne Hospital, Parkville, VIC 3050 Australia; 218 Medical Oncology and Clinical Haematology Unit, Western Hospital, Footscray, VIC 3011 Australia; 219 Medical Psychology Research Unit, Room 332, Brennan MacCallum Building (A18), The University of Sydney, Camperdown, NSW 2006 Australia; 220 Westmead Institute for Cancer Research, Westmead Millennium Institute, Westmead, NSW 2145 Australia; 221 Kolling Institute of Medical Research, Royal North Shore Hospital, St Leonards, NSW 2065 Australia; 222 Department of Oncology, St Vincent’s Hospital, 41 Victoria Parade, Fitzroy, VIC 3065 Australia; 223 Centro Nacional de Investigaciones Oncologicas, C/ Melchor Fernández Almagro, 3E-28029 Madrid, Spain; 224 Western Health and Peter MacCallum Cancer Centre, St Andrew’s Place, East Melbourne, Victoria 3002 Australia; 225 Southern Health Familial Cancer Centre, Special Medicine Building, 246 Clayton Road, Clayton, Vic 3168 Australia; 226 Genetic Services of Western, Level 3 Agnes Walsh House, 374 Bagot Road, Subiaco, WA 6008 Australia; 227 Tumour Development Group, Garvan Institute of Medical Research, The Kinghorn Cancer Centre, 370 Victoria St, Darlinghurst, NSW 2010 Australia; 228 Familial Cancer and Clinical Genetics, Royal Melbourne Hospital, Grattan Street, Parkville, VIC 3050 Australia; 229 Tas Clinical Genetics Service, Royal Hobart Hospital, GPO Box 1061, Hobart, Tasmania 7001 Australia; 230 The Gene Council, Perth, Australia, PO Box 510, North Perth, WA 6906 Australia; 231 Department of Medical Oncology, Peter MacCallum Cancer Centre, St Andrew’s Place, East Melbourne, VIC 3002 Australia; 232 Parkville Familial Cancer Centre and Genomic Medicine, VCCC Grattan Street, Melbourne, Vic 3000 Australia; 233 Familial Cancer centre, Westmead Hospital, Westmead, NSW 2145 Australia; 234 Oncology Service, Christchurch Hospital, Private Bag, 4710 Christchurch, New Zealand; 235 Centre for Genetic Education, Prince of Wales Hospital, Randwick, NSW 2031 Australia; 236 Anatomical Pathology, UNSW, Prince of Wales Hospital, Randwick, 2031 NSW Australia; 237 School of Surgery and Pathology, QE11 Medical Centre, M block, 2nd Floor, Nedlands, WA 6907 Australia; 238 Breast Pathology, University of Queensland, Centre for Clinical Research, Building 71/918, Royal Brisbane and Women’s Hospital, Herston, Qld 4029 Australia; 239 Hunter Area Pathology Service, John Hunter Hospital, Locked Bag 1 Regional Mail Centre, New Lambton Heights, NSW 2310 Australia; 240 Research Department, WEHI c/o Royal Melbourne Hospital, Parkville, VIC 3050 Australia; 241 Familial Cancer Centre, Royal Melbourne Hospital, Grattan Street, Parkville, Vic 3050 Australia; 242 Obstetrics and Gynaecology, University of Auckland, Auckland, New Zealand; 243 The University of Queensland, Building 71/918, RBWH Campus, Herston, Qld 4029 Australia; 244 Genetic Epidemiology Laboratory, Departemnt of Pathology, University of Melbourne, Melbourne, VIC 3010 Australia; 245 Cancer Unit, Queensland Institute of Medical Research, Herston, QLD 4029 Australia; 246 Familial Cancer and Genetics Medicine, Royal Melbourne Hospital, 2nd Floor, Grattan Street, Parkville, Vic 3050 Australia; 247 Cancer Program, Monash University, Rm 349, Level 3, Building 7619, Innovation Walk, Clayton, VIC 3800 Australia; 248 Research Department, Peter MacCallum Cancer Centre, St Andrew’s Place, East Melbourne, VIC 3002 Australia; 249 University of NSW, Prince of Wales Hospital, Barker Street, Randwick, NSW 2031 Australia; 250 Heredity Cancer Clinic, Prince of Wales Hospital, Randwick, NSW 2031 Australia; 251 The Walter and Eliza Hall Institute of Medical Research, Post Office Royal Melbourne Hospital, Parkville, VIC 3050 Australia; 252 Molecular Cancer Epidemiology Laboratory, Queensland Institute of Medical Research, P.O. Royal Brisbane Hospital, Herston, Qld 4027 Australia; 253 Family Cancer Clinic, St Vincent’s Hospital, Darlinghurst, NSW 2010 Australia; 254 Department of Genetics, Royal Melbourne Hospital, Parkville, VIC 3050 Australia; 255 Genome.One, 370 Victoria St, Darlinghurst, 2010 NSW Australia

**Keywords:** Cancer epidemiology, Cancer genetics, Breast cancer

## Abstract

**Background:**

Epidemiological studies provide strong evidence for a role of endogenous sex hormones in the aetiology of breast cancer. The aim of this analysis was to identify genetic variants that are associated with urinary sex-hormone levels and breast cancer risk.

**Methods:**

We carried out a genome-wide association study of urinary oestrone-3-glucuronide and pregnanediol-3-glucuronide levels in 560 premenopausal women, with additional analysis of progesterone levels in 298 premenopausal women. To test for the association with breast cancer risk, we carried out follow-up genotyping in 90,916 cases and 89,893 controls from the Breast Cancer Association Consortium. All women were of European ancestry.

**Results:**

For pregnanediol-3-glucuronide, there were no genome-wide significant associations; for oestrone-3-glucuronide, we identified a single peak mapping to the *CYP3A* locus, annotated by rs45446698. The minor rs45446698-C allele was associated with lower oestrone-3-glucuronide (−49.2%, 95% CI −56.1% to −41.1%, *P* = 3.1 × 10^–18^); in follow-up analyses, rs45446698-C was also associated with lower progesterone (−26.7%, 95% CI −39.4% to −11.6%, *P* = 0.001) and reduced risk of oestrogen and progesterone receptor-positive breast cancer (OR = 0.86, 95% CI 0.82–0.91, *P* = 6.9 × 10^–8^).

**Conclusions:**

The CYP3A7*1C allele is associated with reduced risk of hormone receptor-positive breast cancer possibly mediated via an effect on the metabolism of endogenous sex hormones in premenopausal women.

## Background

Epidemiological studies provide strong evidence for a role of endogenous hormones in the aetiology of breast cancer.^1,2^ Pooled analyses of data from prospective studies estimated that a doubling of circulating oestradiol or oestrone was associated with a 30–50% increase in breast cancer risk in postmenopausal women and a 20–30% increase in breast cancer risk in premenopausal women; there was no evidence that premenopausal progesterone levels were associated with breast cancer risk.^2,3^ We have previously screened 642 SNPs tagging 42 genes involved in sex steroid synthesis or metabolism, and tested for the association with premenopausal urinary oestrone glucuronide and pregnanediol-3-glucuronide levels, measured in urine samples collected at pre-specified days of the woman’s menstrual cycle.^4^ Oestrone-3-glucuronide and pregnanediol-3-glucuronide are urinary metabolites of oestrogen and progesterone, respectively,^5,6^ that are used in the context of reproductive medicine to monitor ovarian activity.^7^ None of the variants that we tested was associated with urinary pregnanediol-3-glucuronide, but a rare haplotype, defined by two SNPs spanning the cytochrome P450 family 3 subfamily A (*CYP3A*) gene cluster, was associated with a highly significant 32% difference in urinary oestrone-3-glucuronide.^4^ Fine-scale mapping analyses identified the SNP rs45446698 as a putative causal variant at this locus; rs45446698 is one of seven highly correlated SNPs that cluster within the *CYP3A7* promoter and comprise the CYP3A7*1C allele.^8^ A genome-wide association study (GWAS) of postmenopausal plasma oestradiol levels found no association at this locus.^9^ A subsequent GWAS of pre- and postmenopausal hormone levels similarly found no association with plasma oestradiol at this locus; they did however find associations at this locus with DHEAS and progesterone.^10^

The *CYP3A* genes (*CYP3A5*, *CYP3A7* and *CYP3A4*) encode enzymes that metabolise a diverse range of substrates;^11^ in addition to a role in the oxidative metabolism of hormones, CYP3A enzymes metabolise ~50% of all clinically used drugs, including many of the agents used in treating cancer.^12^ CYP3A4, the major isoform in adults, is predominantly expressed in the liver, where it is the most abundant P450, accounting for 30% of total CYP450 protein. *CYP3A7*, the major isoform in the foetus, is generally silenced shortly after birth.^13^ In CYP3A7*1C carriers, a region within the foetal *CYP3A7* promoter has been replaced with the equivalent region from the adult *CYP3A4* gene;^14^ this results in adult expression of *CYP3A7* in CYP3A7*1C carriers and may influence metabolism of endogenous hormones, exogenous hormones used in menopausal hormone treatment and clinically prescribed drugs, including agents used in treating cancer, in these individuals.^12,15^ In order to identify additional variants that are associated with premenopausal urinary hormone levels and to further characterise the associations at the *CYP3A* locus, we carried out a GWAS of urinary oestrone-3-glucuronide and pregnanediol-3-glucuronide levels, using mid-luteal-phase urine samples from women of European ancestry and followed up by testing for an association with breast cancer risk in cases and controls from the Breast Cancer Association Consortium (BCAC). To determine whether the CYP3A7*1C allele influences metabolism of exogenous hormones, we evaluated gene-environment interactions with menopausal hormone treatment for breast cancer risk, and to investigate whether adult expression of *CYP3A7* impacts on agents used in treating cancer, we analysed associations with breast cancer-specific survival.

## Methods

### GWAS subjects

#### Generations Study

Full details of the Generations Study have been published previously.^16^ Briefly, the Generations Study is a cohort study of more than 110,000 women from the UK general population, who were recruited beginning in 2003 and from whom detailed questionnaires and blood samples have been collected to investigate risk factors for breast cancer.

#### British Breast Cancer Study

Full details of the British Breast Cancer Study have been published previously.^17^ Briefly, the British Breast Cancer Study is a national case–control study of breast cancer, in which cases of breast cancer were ascertained through the cancer registries of England and Scotland and through the National Cancer Research Network. Cases were asked to invite a healthy female first-degree relative with no history of cancer and a female friend or non-blood relative to participate in the study.

#### Mammography Oestrogens and Growth Factors study

Full details of the Mammography Oestrogens and Growth Factors study have been published previously.^18^ Briefly, this is an observational study nested within a trial of annual mammography screening in young women that was conducted in Britain.^19^ Approximately 54,000 women aged 39–41 years were randomly assigned to the intervention arm from 1991 to 1997 and offered annual mammograms until age 48 years. From 2000 to 2003, women in the intervention arm who were still participating in this trial were invited to participate in the Mammography Oestrogens and Growth Factors study; they were asked to provide a blood sample and complete a questionnaire detailing demographic, lifestyle and reproductive factors. More than 8000 women were enrolled in the study.

GWAS subjects were drawn from the Generations Study (*N* = 184), the British Breast Cancer Study (*N* = 284) and the Mammography Oestrogens and Growth Factors study (*N* = 109). To be eligible for the GWAS analysis of oestrone-3-glucuronide and pregnanediol-3-glucuronide levels, women had to be having regular menstrual cycles (i.e., their usual cycle length had to be between 21 and 35 days) and not using menopausal hormone therapy or oral contraceptives. All of the women included in this analysis reported being of European ancestry, and none had been diagnosed with breast cancer at the time of study recruitment.

### Measurement of hormone levels

The protocol for collecting timed urine samples has been published previously.^18^ Briefly, a woman’s predicted date of ovulation was estimated from the date of the first day of her last menstrual period and her usual cycle length; ovulation was predicted to occur 14 days before the date of her next menstrual period. On this basis, women were asked to provide a series of early morning urine samples on pre-specified days of their cycle. For this analysis, the mid-luteal-phase sample, taken at 7 days after the predicted day of ovulation, was used. To confirm that ovulation had occurred, consistent with the predicted date of ovulation, pregnanediol-3-glucuronide was measured; to take account of the differences in volume in early morning urine samples from different women, we measured creatinine, a waste product of normal muscle and protein metabolism that is released at a constant rate by the body. Samples in which pregnanediol-3-glucuronide, adjusted for creatinine levels, was >0.3 µmol/mol, were taken forward for measurement of creatinine-adjusted oestrone-3-glucuronide. Pregnanediol-3-glucuronide and oestrone-3-glucuronide were analysed by commercial competitive ELISA Kits (Arbor Assays, Ann Arbor, USA) according to the manufacturer’s instructions. For pregnanediol-3-glucuronide, the lower limit of detection was determined as 0.64 nmol/l; intra- and inter-assay coefficients of variation were 3.7% and 5.2%, respectively. For oestrone-3-glucuronide, the lower limit of detection was determined as 19.6 pmol/l; intra- and inter-assay coefficients of variation were 3.5% and 5.9%, respectively. Creatinine was determined using the creatininase/creatinase-specific enzymatic method^20^ using a commercial kit (Alpha Laboratories Ltd. Eastleigh, UK) adapted for use on a Cobas Fara centrifugal analyser (Roche Diagnostics Ltd, Welwyn Garden City, UK). For within-run precision, the coefficient of variation was <3%, while for intra-batch precision, the coefficient of variation was <5%.

For 303 premenopausal women participating in the Generations Study (184 as above and an additional 119 for whom timed urine samples were accrued more recently), urinary progesterone levels were also measured using an “in house” ELISA. In all, 96-well plates (Greiner Bio-One GmbH, Frickenhausen, Germany) were coated with 100 µl of 5 µg/ml GAM (Arbor Assays, Ann Arbor, USA) in ELISA coating buffer (100 mM Na Bicarbonate, pH 9.6) covered and incubated in a fridge at 4 °C overnight. Before use, the plates were washed three times with wash buffer 0.05 M Tris/HCl and 0.05% Tween 20, pH 7.4 (Tween^®^ 20, Sigma-Aldrich, Inc., St. Louis, MO, USA). Standards, samples and controls (20 µl per well) were added to each well, followed by 80 µl of progesterone 3-HRP conjugate (Astra Biotech GmbH, Berlin, Germany) at 1:10,000 in assay buffer (PBS pH 7.4 containing 0.1% BSA and 250 ng/ml Cortisol), followed by 50 μl of monoclonal progesterone Ab (Astra Biotech GmbH, Berlin, Germany) 1:50,000 in assay buffer. Plates were incubated at room temperature for 2 h on a microtitre plate shaker (IKA^®^, Schuttler MTS4, IKA Labortechnik, Staufen, Germany), then washed five times with assay wash buffer and 120 µl of substrate solution (3,3,5,5-tetramethylbenzidine, Millipore Corporation, Temecula, CA, USA) was added to each well. Plates were incubated at room temperature without shaking in the dark. After 20 min, the reaction was stopped by adding 80 µl of 2 N H_2_SO_4_ solution (Sigma-Aldrich Company Ltd., Dorset, UK). Finally, the plates were read on a plate reader at 450 nm. Standard curves were prepared with a total of eight different concentrations (16, 8, 4, 2, 1, 0.5, 0.25 and 0 ng/ml). Samples, standards and controls were included in duplicate. Inter- and intra-assay coefficients of variation were calculated from two controls of low and high progesterone in duplicate in each of eight assays. The inter-assay coefficients of variation for low and high pools, respectively, were 11.4 and 9.1%; the intra-assay coefficients of variation were 8.9 and 5.6%. The lower limit of detection was calculated at 0.1 ng/ml. Cross-reaction with other steroids was oestrone: 0.17%, oestradiol: 0.28%, oestriol: 0.18%, dehydroepiandrosterone: 0.02%, testosterone: 0.36%, dihydrotestosterone: 0.15%, 17α-hydroxyprogesterone: 2.9%, androstenedione: 0.14%, 11-deoxycortisol: 0.46%, corticosterone: 0.18%, cortisone: 0.04% and cortisol: 0.04%.

### GWAS genotyping and quality control

DNA from 577 women was genotyped using Illumina Infinium OncoArray 500 K BeadChips. We excluded samples for which <95% of SNPs were successfully genotyped. Identity-by-descent analysis was used to identify closely related individuals enabling exclusion of first-degree relatives. We applied SmartPCA^21^ to our data and used phase II HapMap samples to identify individuals with non-Caucasian ancestry. The first two principal components for each individual were plotted, and k-means clustering was used to identify samples separated from the main Caucasian cluster. SNPs with call rates <95% were excluded, as were SNPs with minor allele frequency (MAF) < 2% and those whose genotype frequencies deviated from Hardy–Weinberg proportions at *P* < 1 × 10^–05^. Following QC, 487,659 SNPs were successfully genotyped in 560 samples (Generations Study: *N* = 179, British Breast Cancer Study: *N* = 278 and Mammography Oestrogens and Growth Factors study: *N* = 103). Genome-wide imputation was performed using 1KGP Phase 3 reference data. Haplotypes were pre-phased using SHAPEIT2.^22^ Imputation was performed using IMPUTE2.^23^ Imputed SNPs with INFO scores <0.8 and MAFs <2% were excluded from subsequent analyses. After QC, a set of 7,792,694 successfully imputed SNPs were available for association analysis.

### Genotyping rs45446698 and sequencing of the CYP3A7*1C allele

For the 119 Generations Study women who were not included in the GWAS but for whom progesterone was subsequently measured, rs45446698 was genotyped by TaqMan (Thermo Fisher Scientific Ltd, UK). The call rate was 100% with 100% concordance between 12 duplicates. To confirm that rs45446698 tags the CYP3A7*1C allele, we sequenced this region in 31 women selected on the basis of their rs45446698 genotype (9 common homozygotes and 22 carriers). A 370-bp DNA region (chr7: 99 332 745-99 333 114; GRCh37/hg19) was amplified using Phusion High-Fidelity DNA Polymerase (New England Biolabs, UK) and primers CCATAGAGACAAGAGGAGA (forward) and CTGAGTCTTTTTTTCAGCAGC (reverse). The PCR product was purified using QIAquick Gel Extraction Kit (Qiagen) and Sanger sequenced using a commercially available service (Eurofins Genomics, Germany).

### Statistical analysis of GWAS data

Tests of association between SNP genotypes and log-transformed creatinine-adjusted oestrone-3-glucuronide and pregnanediol-3-glucuronide adjusted for study were performed using linear regression in SNPTEST v2.5.^24^ Test statistic inflation was assessed visually using a QQ Plot (Supplementary Fig. S1) and formally by calculating the inflation factor, *λ*. There was no evidence of systematic test statistic inflation (*λ* = 1.01 for both oestrone-3-glucuronide and pregnanediol-3-glucuronide). For the single significant association (rs45446698), we used multivariate linear regression to adjust for potential confounders: age at menarche (<12, 12, 13, 14 and >14 years), age at collection of urine samples (<35, 35–40 and ≥40 years), body mass index (BMI: < 18.5, 18.5–<20.0, 20.0–<25.0, 25.0–<30.0 and ≥ 30.0 kg/m^2^) and parity (0, 1, 2 and ≥3 live births).

### Follow-up genotyping of rs45446698

Genotype data for rs45446698 were generated as part of iCOGS^25^ and OncoArray.^26^ Full details of SNP selection, array design, genotyping and post-genotyping QC have been published.^25,26^ Participants genotyped in both collaborations were excluded from the iCOGS data sets with the exception of the GxE interaction analysis of menopausal hormone treatment, for which five studies (CPS-II, PBCS, UKBGS, MCCS and pKARMA) were excluded from OncoArray, rather than iCOGS, in order to maximise the number of studies with sufficient cases and controls for analysis. We excluded cases with breast tumours of unknown invasiveness, or in situ disease, and those for whom age at diagnosis was not known. After QC exclusions,^26^ the call rate for rs45446698 in OncoArray data was 99.66% and there was no evidence of deviation from Hardy–Weinberg equilibrium in controls (Supplementary Table S1). In iCOGS data, rs45446698 was imputed using 1KGP Phase 3 reference data (info score = 0.94); we used gene dosages (≤0.2 = 0, >0.8 and ≤1.2 = 1, >1.8 = 2) to call genotypes for 99.22% of samples.

### Statistical analysis of rs45446698 and breast cancer risk

Due to the low MAF of rs45446698 (3.7%, 0.03% and 0.4% in individuals of European, Asian and African ancestry, respectively), we restricted our analyses to individuals of European ancestry and excluded studies with <50 cases or controls; there were 35 (iCOGS) and 56 (OncoArray) studies for the current case–control analysis (Supplementary Tables S1 and S2).

We combined heterozygote and rare homozygote genotypes and estimated carrier ORs using logistic regression, adjusted for 15 principal components^25,26^ and study. Stratum-specific carrier ORs were estimated for a set of pre-specified prognostic variables (oestrogen receptor (ER), progesterone receptor (PR), HER2, grade and stage). We excluded studies with <50 cases or controls in any individual stratum from stratified analyses. Interactions were assessed based on case-only models (ER, PR, Her2, stage and grade). In the subset of studies for which covariate data were available, we used multivariable logistic regression to adjust for reference age (defined as age at diagnosis for cases and age at interview for controls), age at menarche, BMI and parity (as above). Finally, we stratified our analyses on menopausal status at reference age. When menopausal status was missing, the reference age was used as a surrogate (<54 premenopausal and ≥54 postmenopausal). To select the reference age that most accurately captured menopausal status in this group of studies, we generated AUC curves based on women who had reported natural menopause with different reference age cut-offs (50–56 years); on this basis, a reference age of 54 was selected. *P* values were estimated using likelihood ratio tests with one degree of freedom. All *P* values reported, for all analyses, are two-sided. Statistical analyses were performed using STATA version 11.0 (StataCorp, College Station, TX, USA).

### Statistical analysis of gene-environment interaction (GxE) with menopausal hormone treatment

Postmenopausal women from 13 (iCOGS) and 27 (OncoArray) studies provided the data on menopausal hormone treatment. Menopausal status and postmenopausal hormone use were derived as of the reference date (defined as date of diagnosis for cases and interview for controls); women with unknown age at reference date were excluded from this analysis. All analyses were conducted only in postmenopausal women. Carrier ORs for breast cancer risk were estimated using logistic regression stratified by current use of menopausal hormone treatment, oestrogen–progesterone therapy and oestrogen-only therapy, respectively. Analyses were adjusted for study, ten principal components, reference age, age at menarche, parity, BMI, former use of menopausal hormone treatment and use of any menopausal hormone treatment preparation other than the one of interest in analyses of current use of menopausal hormone treatment by type. To account for potential heterogeneity of the main effects of menopausal hormone treatment/oestrogen–progesterone therapy/oestrogen-only therapy by study design, we included an interaction term between the risk factor of interest and an indicator variable for study design (prospective cohorts/population-based case–control studies, non-population-based studies). Interactions between rs45446698 and current use of menopausal hormone treatment, oestrogen–progesterone therapy and oestrogen-only therapy were assessed using likelihood ratio tests, based on logistic regression models with and without interaction between rs45446698 and current use of menopausal hormone treatment, oestrogen–progesterone therapy and oestrogen-only therapy, respectively. Statistical analyses were performed using SAS 9.4 and R (version 3.4.4).

### Statistical analysis of breast cancer-specific survival in cases

In total, 38 (iCOGS) and 63 (OncoArray) studies provided follow-up data for analysis of breast cancer-specific survival. Analysis of outcome was restricted to patients who were at least 18 years old at diagnosis and for whom vital status at, and date of the last follow-up were known. Patients ascertained for a second tumour were excluded. Time-to-event was calculated from the date of diagnosis. For prevalent cases with study entry after diagnosis, left truncation was applied, i.e., follow-up started at the date of study entry.^27^ Follow-up was right-censored at the date of death (death known to be due to breast cancer considered an event), the date the patient was last known to be alive if death did not occur or at 10 years after diagnosis, whichever came first. Follow-up was censored at 10 years due to limited data availability after this time. Hazard ratios (HR) for association of rs45446698 genotype with breast cancer-specific survival were estimated using Cox proportional hazards regression implemented in the R package survival (v. 2.43–3) stratified by country. iCOGS and OncoArray estimates were combined using an inverse-variance-weighted meta-analysis.

## Results

**Fig. 1:**
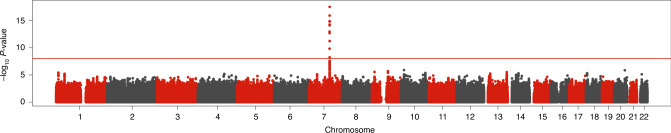
Manhattan plot of single-nucleotide polymorphism (SNP) associations with luteal-phase urinary oestrone-3-glucuronide levels in 560 premenopausal women. –log_10_ *P* values for SNP associations are plotted against the genomic coordinates (hg19). The red line indicates the conventionally accepted threshold for genome-wide significance (*P* = 1 × 10^−8^).

We tested 8,280,353 autosomal SNPs for association with luteal-phase creatinine-adjusted oestrone-3-glucuronide and pregnanediol-3-glucuronide in 560 premenopausal women. For oestrone-3-glucuronide, we identified a single peak mapping to the *CYP3A* locus at chromosome 7q22.1 (Fig. 1 and Supplementary Table S3); conditioning on any of the top SNPs, there were no additional independent signals. Four of the SNPs that were significant at *P* < 1 × 10^−8^ comprise part of the seven-SNP CYP3A7*1C allele,^8,15^ including the top, directly genotyped SNP, rs45446698 (Supplementary Table S3). The rare rs45446698-C allele (MAF = 0.035) was associated with a 49.2% reduction in luteal-phase oestrone-3-glucuronide (95% CI −56.1% to −41.1%, *P* = 3.1 × 10^−18^, Table 1) and explained 11.5% of the variation in oestrone-3-glucuronide in these premenopausal women. Since hormone levels may be influenced by both demographic and reproductive factors, we adjusted for age at urine collection, age at menarche, body mass index and parity; these adjustments did not alter the association (fully adjusted model: 44.8% reduction, 95% CI −53.3% to −34.8%, *P* = 2.1 × 10^−12^, Table 1).

**Table 1:** Association of rs45446698 with levels of oestrone-3-glucuronide, pregnanediol-3-glucuronide and progesterone in premenopausal women of European ancestry.

Hormone	Geometric mean by rs45446698 genotype (µmol/mol)		Unadjusted analysis		Adjusted analysis^a^	
	AA	AC/CC	% change	*P*	% change	*P*
*GWAS (*N *=* *560*)						
Oestrone-3-glucuronide	9.74	4.95	−49.2 (−56.1 to −41.1)	3.1 × 10^–18^	−44.8 (−53.3 to −34.8)	2.1 × 10^–12^
Pregnanediol-3-glucuronide	0.78	0.70	−10.1 (−22.5 to 4.3)	0.16	−9.3 (−22.9 to 6.8)	0.24
*Follow-up progesterone analysis (*N *=* *298)*						
Oestrone-3-glucuronide	9.77	4.39	−55.0 (−63.1 to −45.1)	2.6 × 10^–15^	−55.1 (−63.6 to −44.7)	4.0 × 10^–14^
Pregnanediol-3-glucuronide	0.79	0.75	−5.5 (−24.2 to 17.7)	0.61	−9.6 (−27.9 to 13.3)	0.38
Progesterone	29.37	21.51	−26.7 (−39.4 to −11.6)	0.001	−24.3 (−37.6 to −8.2)	0.005

^a^Analysis was adjusted for age at menarche (<12, 12, 13, 14 and >14 years), age at collection of urine samples (<35, 35–40 and ≥40 years), body mass index (<18.5, 18.5–<20.0, 20.0–<25.0, 25.0–<30.0 and ≥30.0 kg/m^2^) and parity (0, 1, 2 and ≥3 live births).

For pregnanediol-3-glucuronide, there were no associations that were significant at a threshold of *P* < 1 × 10^−8^ (Supplementary Fig. S2). An association between the *CYP3A* locus and progesterone levels has been reported previously;^10^ accordingly, we measured progesterone in addition to pregnanediol-3-glucuronide in premenopausal women from the Generations Study. Progesterone was moderately correlated with pregnanediol-3-glucuronide (*r* = 0.37, *P* = 7.4 × 10^−12^), but while there was no association between the rs45446698-C allele and urinary pregnanediol-3-glucuronide levels (5.5% reduction, 95% CI −24.2% to +17.7%, *P* = 0.61) in this group of women, the rs45446698-C allele was associated with significantly lower luteal-phase urinary progesterone levels (26.7% reduction, 95% CI −39.4% to −11.6%, *P* = 0.001, Table 1). Adjusting these analyses for covariates, as above, did not alter the results (Table 1).

To test for the association between rs45446698 and breast cancer risk, we combined genotype data from 56 studies (OncoArray; Supplementary Table S1) with imputed data from 35 studies (iCOGS; Supplementary Table S2) in a total of 90,916 cases and 89,893 controls of European Ancestry. The rs45446698-C allele was associated with a reduction in breast cancer risk (OR = 0.94, 95% CI 0.91–0.98, *P* = 0.002, Table 2) with no evidence of heterogeneity between data sets (*P*_het_ = 0.58). There was no evidence that the reduction in breast cancer risk associated with being a rs45446698-C carrier differed according to Her2 status, tumour grade or stage (Supplementary Table S4). Stratifying by ER status, the association was limited to ER-positive (ER + ) breast cancers (OR = 0.91, 95% CI 0.87–0.96, *P* = 0.0002 and OR = 1.03, 95% CI 0.95–1.11, *P* = 0.50 for ER + and ER− cancers, respectively; *P*_int_ = 0.03, Table 2). Stratifying by ER and PR status, the association was limited to ER + /PR + cancers (ER + /PR + : OR = 0.86, 95% CI 0.82–0.91, *P* = 6.9 × 10^–8^; ER + /PR−: OR = 1.06, 95% CI 0.96–1.16, *P* = 0.25; *P*_int_ = 0.0001, Table 2). Adjusting for demographic and reproductive factors in the subset of studies for which these additional covariates were available did not alter this association (Supplementary Table S5). Defining reference age as age at diagnosis for cases and age at interview for controls and using this as a proxy for menopausal status (<54 or ≥54 years), we further stratified our analysis on menopausal status; there was little evidence that the association with ER + /PR + breast cancer differed by menopausal status (premenopausal OR = 0.94, 95% CI 0.84–1.06, *P* = 0.31, postmenopausal OR = 0.86, 95% CI 0.80–0.93, *P* = 0.0001, *P*_het_ = 0.28).

**Table 2:** Association of rs45446698 among women of European ancestry overall and stratified by hormone receptor status.

	iCOGS				OncoArray				Combined			
	Cases	Controls	OR (95% CI)	*P*_1_	Cases	Controls	OR (95% CI)	*P*_1_	Cases	Controls	OR (95% CI)	*P*_1_
All subjects	36,859	37,320	0.93 (0.87–0.98)	0.01	54,057	52,573	0.95 (0.91–1.00)	0.05	90,916	89,893	0.94 (0.91–0.98)	0.002
												*P*_het_ = 0.58
ER +	19,950	28,820	0.90 (0.84–0.97)	0.007	28,478	40,223	0.92 (0.87–0.98)	0.01	48,428	69,043	0.91 (0.87–0.96)	0.0002
ER−	4298	28,820	1.04 (0.92–1.18)	0.56	6592	40,223	1.03 (0.92–1.14)	0.64	10,890	69,043	1.03 (0.95–1.11)	0.50
NK	5087				8380							
Total	29,335	28,820		*P*_int_ = 0.06	4450	40,223		*P*_int_ = 0.19				*P*_int_ = 0.03
PR +	13,995	28,820	0.85 (0.78–0.93)	0.0002	21,500	40,223	0.87 (0.81–0.93)	0.0001	35,495	69,043	0.86 (0.82–0.91)	5.8 × 10^–8^
PR−	6154	28,820	1.06 (0.95–1.18)	0.33	10,058	40,223	1.05 (0.96–1.15)	0.26	16,212	69,043	1.05 (0.98–1.12)	0.18
NK	9186				11,892							
Total	29,335	28,820		*P*_int_ = 0.001	43,450	40,223		*P*_int_ = 0.0004				*P*_int_ = 1.3 × 10^–6^
ER + , PR +	13,508	28,820	0.85 (0.78–0.93)	0.0003	20,624	40,223	0.87 (0.81–0.93)	0.0001	34,132	69,043	0.86 (0.82–0.91)	6.9 × 10^–8^
ER + , PR−	2890	28,820	1.03 (0.89–1.20)	0.66	4597	40,223	1.07 (0.96–1.21)	0.21	7487	69,043	1.06 (0.96–1.16)	0.25
NK	12,937				18,229							
Total	29,335	28,820		*P*_int_ = 0.02	43,450	40,223		*P*_int_ = 0.001				*P*_int_ = 0.0001

*P*_1_   test of H_0_ no association between rs45446698 and breast cancer risk, *P*_int_   test of H_0_ no difference between stratum-specific estimates, *P*_het_   test of H_0_ no difference between iCOGS and OnocoArray data, *NK* not known.

Studies with less than 50 cases in any stratum were excluded from the stratified analyses leaving 16 studies for analysis in iCOGS data and 32 studies for analysis in OncoArray data.

On the assumption that genetic variants that influence metabolism of endogenous hormones^5^ may also impact on metabolism of exogenous hormones, we investigated whether menopausal hormone treatment modified the association between rs45446698 genotype and ER + /PR + breast cancer risk in 17,831 postmenopausal breast cancer cases and 40,437 postmenopausal controls. The rs45446698-C carrier OR was lower (i.e., more protective) in current users of any menopausal hormone treatment but particularly in those who used combined oestrogen–progesterone therapy (current users: OR = 0.68, 95% CI 0.52–0.90, *P* = 0.007; never users: OR = 0.85, 95% CI 0.76–0.95, *P* = 0.005, Table 3). This difference was not, however, statistically significant (*P*_int_ = 0.15, Table 3).

**Table 3:** Association of rs45446698 genotype with ER + /PR + breast cancer risk among women of European ancestry stratified by current use of postmenopausal hormone treatment.

	iCOGS				OncoArray				Combined			
	Cases	Controls	OR (95% CI)	*P*_1_	Cases	Controls	OR (95% CI)	*P*_1_	Cases	Controls	OR (95% CI)	*P*_1_
MHT−	3742	8902	0.73 (0.62–0.86)	0.0002	5961	15,128	0.95 (0.83–1.08)	0.45	9703	24,030	0.86 (0.78–0.95)	0.003
MHT +	1593	2859	0.77 (0.59–0.99)	0.04	2823	5529	0.87 (0.71–1.06)	0.16	4416	8388	0.83 (0.70–0.97)	0.02
NK	622	1793			3090	6226			3712	8019		
Total	5957	13,554		*P*_int_ = 0.81	11,874	26,883		*P*_int_ = 0.26	17,831	40,437		*P*_int_ = 0.47
EPT−	3736	7826	0.77 (0.65–0.91)	0.002	4173	8566	0.93 (0.80–1.09)	0.38	7909	16,392	0.85 (0.76–0.95)	0.005
EPT +	727	944	0.71 (0.48–1.05)	0.09	878	1170	0.66 (0.45–0.97)	0.03	1605	2114	0.68 (0.52–0.90)	0.007
NK	1494	4784			6823	17,147			8317	21,931		
Total	5957	13,554		*P*_int_ = 0.72	11,874	26,883		*P*_int_ = 0.09	17,831	40,437		*P*_int_ = 0.15
ET−	3840	7710	0.75 (0.63–0.88)	0.0005	4343	8136	0.92 (0.79–1.07)	0.29	8183	15,846	0.88 (0.79–0.97)	0.01
ET +	589	1172	0.69 (0.45–1.06)	0.09	640	1484	0.94 (0.62–1.42)	0.76	1229	2656	0.84 (0.64–1.10)	0.21
NK	1528	4672			6891	17,263			8419	21,935		
Total	5957	13,554		*P*_int_ = 0.83	11,874	26,883		*P*_int_ = 0.85	17,831	40,437		*P*_int_ = 0.78

*MHT*    menopausal hormone treatment, *EPT*   oestrogen–progesterone therapy, *ET*   oestrogen-only therapy, *P*_1_   test of H_0_ no association between rs45446698 and ER + /PR + breast cancer risk, *P*_int_   test of H_0_ no difference between stratum-specific estimates, *NK* not known.

Studies with less than 50 cases in any stratum were excluded from the stratified analyses leaving 13 studies for analysis in iCOGS data and 27 studies for analysis in OncoArray data. All models are adjusted for reference age, study, ten principal components and former use of MHT. Additionally, when stratified by EPT or ET, models are adjusted for use of any other type of MHT other than the one of interest. Further adjusting for age at menarche (<12, 12, 13, 14, >14), parity (0, 1, 2 and ≥3 live births) and BMI ( < 18.5, 18.5–<20.0, 20.0–<25.0, 25.0–<30.0 and ≥30.0  kg/m^2^) did not alter these results.

Finally, to determine whether rs45446698 genotype could affect patient outcome by influencing metabolism of cytotoxic agents that are CYP3A substrates,^15^ we tested for the association between rs45446698 genotype and 10-year breast cancer-specific survival in 91,539 breast cancer cases from 71 studies for whom follow-up data were available. There was neither overall association between rs45446698 genotype and breast cancer-specific survival (HR = 0.99, 95% CI 0.91–1.09, *P* = 0.90, Table 4) nor was there any evidence of an association in analyses stratified by tumour characteristics (Supplementary Table S6). Stratifying by treatment regimen, we found no evidence that rs45446698 genotype influenced outcome in cases who were treated with a hormonal agent (i.e., tamoxifen or an aromatase inhibitor, Table 4). There was, however, some evidence that in cases who were treated with a taxane, carriers of the rs45446698-C allele had reduced breast cancer-specific survival compared with non-carriers (HR = 1.46, 95% CI 1.08–1.97, *P* = 0.01, Table 4).

**Table 4:** Association of rs45446698 with breast cancer-specific survival in breast cancer cases of European Ancestry stratified by treatment regimen.

Group	iCOGS				OncoArray				Combined		
	Cases	Events	HR (95% CI)	*P*_1_	Cases	Events	HR (95% CI)	*P*_1_	HR (95% CI)	*P*_1_	*P*_het_
All breast cancer patients	32,743	2580	0.93 (0.80–1.08)	0.35	58,796	3799	1.04 (0.92–1.17)	0.57	0.99 (0.91–1.09)	0.90	0.28
Only patients that											
Received tamoxifen	9766	825	1.22 (0.95–1.57)	0.13	7803	746	0.95 (0.73–1.23)	0.68	1.08 (0.90–1.30)	0.41	0.18
Received aromatase inhibitor	3794	246	0.94 (0.58–1.54)	0.82	5460	247	1.03 (0.64–1.65)	0.91	0.99 (0.70–1.39)	0.94	0.81
Received CMF-like CT	919	99	0.30 (0.09–1.01)	0.05	1692	229	0.88 (0.55–1.41)	0.60	0.77 (0.50–1.19)	0.24	0.11
Received taxanes*	1806	160	1.69 (0.96–2.99)	0.07	3836	299	1.37 (0.96–1.96)	0.08	1.46 (1.08–1.97)	0.01	0.54
Received anthracycline therapy	4625	418	1.21 (0.83–1.75)	0.32	6740	771	1.07 (0.82–1.38)	0.63	1.11 (0.90–1.37)	0.33	0.58

*CMF*   cyclophosphamide methotrexate fluorouracil, *CT*   chemotherapy, *P*_1_   test of H_0_ no association between rs45446698 and breast cancer-specific survival, *P*_het_   test of H_0_ no difference across genotyping platforms.

In total, 38 studies from iCOGS and 63 studies from OnocoArray provided follow-up data for analysis of breast cancer-specific survival. The results were censored at 10 years after diagnosis. HR for association of rs45446698 genotype with breast cancer-specific survival was estimated using Cox proportional hazards regression stratified by country.

*To test for statistical interaction between rs45446698 genotype and treatment with a taxane, we additionally compared the association in cases who received chemotherapy including a taxane to that in cases who received chemotherapy that did not include a taxane (*P*_int_ = 0.02; the association in the latter group was in the opposite direction and not significant: HR = 0.88, 95% CI 0.67–1.15, *P* = 0.34).

## Discussion

This present GWAS identified a single, highly significant association between the CYP3A7*1C allele (tagged by rs4546698) and premenopausal urinary oestrone-3-glucuronide. This finding alone is not novel; we have previously reported an association between the CYP3A7*1C allele, parent oestrogens and several oestrogen metabolites.^5^ What we have demonstrated for the first time is the extent to which this signal dominates the genetic architecture of hormone levels in premenopausal women of Northern European ancestry (Fig. 1; rs45446698 *P* = 3.1 × 10^−18^, all other signals *P* > 1 × 10^–8^) and we estimate that 11.5% of the variance in urinary oestrone-3-glucuronide levels is explained by this one allele.

Two previous GWAS of circulating oestrogen levels have been published, neither reported an association with the *CYP3A* locus.^9,10^ This lack of replication may be explained by our choice of study population. The first GWAS^9^ was conducted in postmenopausal women (*N* = 1623) participating in the Nurses’ Health Study and the Sisters in Breast Screening Study. The second was conducted within the Twins UK study (*N* = 2913) and included men as well as pre-, peri- and postmenopausal women. A strength of our GWAS is that all of the women were premenopausal and had regular menstrual cycles; circulating levels of oestrogens in premenopausal women are much higher compared with those in postmenopausal women.^4,28^ For each woman, we assayed a single urine sample taken in the mid-luteal phase of her cycle at exactly 7 days after her predicted day of ovulation. Thus, although our study is relatively small (*N* = 560), we may have had greater power to detect an association at the *CYP3A* locus than previous studies due to the very homogeneous premenopausal study population that we selected.

Our findings also demonstrate the potential significance of the choice of hormone or hormone metabolite; both of the previous GWAS assayed plasma oestradiol. In a targeted analysis of urinary oestrogen metabolites, we have previously shown that the association between the CYP3A7*1C allele and oestrone (45.3% lower levels in carriers, *P* = 0.0005) is more pronounced than the association with oestradiol (26.7% lower levels, *P* = 0.07) with the implication that measuring urinary oestrone-3-glucuronide (rather than plasma oestradiol) may have contributed to our positive findings. Similarly, by measuring pregnanediol-3-glucuronide and progesterone in premenopausal women from the Generations Study, we were able to demonstrate a significant association of rs45446698 with progesterone (27% reduction, *P* = 0.001) in the absence of an association with pregnanediol-3-glucuronide (6% reduction, *P* = 0.61).

The fact that we measured a urinary oestrogen metabolite (oestrone-3-glucuronide) rather than serum or plasma oestrogens (oestradiol or oestrone) limits the interpretation of our results in terms of a causal association. Estimates of the association between circulating oestrogens and breast cancer risk are based on measurements of hormone levels in plasma or serum,^3^ and in a recent study that measured luteal-phase serum oestrogens and urinary oestrogen metabolites in 249 premenopausal women,^29^ serum oestradiol and oestrone were only moderately correlated with urinary oestrone (serum oestradiol: *r* = 0.39, serum oestrone: *r* = 0.48). Our analysis of rs45446698 genotypes in 90,916 cases and 89,893 controls from BCAC, however, provides robust evidence of an association of the CYP3A7*1C allele with breast cancer risk overall (OR = 0.94, *P* = 0.002) and a more pronounced protective effect on ER + /PR + breast cancers (OR = 0.86, *P* = 6.9 × 10^−8^). The specificity of this association (comparing ER + /PR− with ER + /PR + cancers, *P*_het_ = 0.001) and our replication of Ruth and colleagues report of a signal at the *CYP3A* locus in their analysis of circulating progesterone levels^10^ raise the possibility that premenopausal progesterone levels might influence risk of ER + /PR + breast cancers. This would be in contrast to the findings from Key et al. who reported no evidence of an association between premenopausal progesterone levels and breast cancer risk overall and no heterogeneity in estimates stratified by PR status.^3^ However, the number of cases of PR + (*N* = 158) and PR− (*N* = 61) breast cancer was small, and this analysis may have lacked power to detect modest associations in subgroups of cancers. Alternatively, the association of rs45446698 genotype with ER + /PR + breast cancer risk, specifically, may be due to the fact that PR is a marker for an intact oestrogen signalling pathway^30^ confirming a direct link between the levels of oestrogen (or oestrogen signalling) and proliferation in this subgroup of cancers.

Our analysis of the CYP3A7*1C allele, menopausal hormone treatment and breast cancer risk was inconclusive; while the carrier ORs were consistent with a greater protective effect of this allele in women taking exogenous hormones, particularly oestrogen–progesterone therapy, none of the interactions was statistically significant. Overall, there were 14,119 ER + /PR + breast cancer cases and 32,418 controls for this subgroup analysis, but for what was, arguably, the most pertinent subgroup (i.e., current oestrogen–progesterone therapy use), the number of cases who were current users was relatively small (CYP3A7*1C carriers *N* = 107, non-carriers *N* = 1498) and power was limited to detect modest interactions. There are limitations to this analysis; we focussed on current menopausal hormone treatment use (adjusted for past use) as it is for current use that the association with breast cancer risk is the strongest,^31^ but we did not have information on dose, duration or the formulation that was used.

Finally, we found no association between CYP3A7*1C carrier status and survival in patients treated with tamoxifen, a known CYP3A substrate. This may reflect the fact that compared to CYP3A4, CYP3A7 is a poor metaboliser of tamoxifen,^32^ or that standard doses of tamoxifen achieve high levels of oestrogen receptor saturation.^33^ There was some evidence that breast cancer-specific survival was reduced in CYP3A7*1C carriers who were treated with a taxane, compared with non-carriers (*P* = 0.01); this may, however, be a chance finding given the number of comparisons that were tested.

In conclusion, we present strong evidence that the CYP3A7*1C allele impacts on the metabolism of endogenous hormones, which in turn, reduces the risk of hormone receptor-positive breast cancer in carriers. Optimal strategies for breast cancer prevention in women at high risk of breast cancer and in the general population are an area of active research. In this context, CYP3A7*1C carriers represent a naturally occurring cohort in which the effects of reduced exposure to endogenous oestrogens and progesterones throughout a woman’s premenopausal years can be further investigated. Our results regarding the impact of CYP3A7*1C carrier status on exogenous hormones and chemotherapeutic agents are preliminary but warrant further investigation, preferably in the setting of randomised trials.

## Supplementary information

Supplemental material

## Data Availability

GWAS data and the complete dataset for follow-up genotyping will not be made publicly available due to restraints imposed by the ethics committees of individual studies; requests for data can be made to the corresponding author (GWAS data) or the Data Access Coordination Committee (follow-up genotyping data) of BCAC (http://bcac.ccge.medschl.cam.ac.uk/). Summary results for all variants genotyped by BCAC (including rs45446698) are available at http://bcac.ccge.medschl.cam.ac.uk/.
