# Supplementary material for: CYP3A7*1C allele: linking premenopausal oestrone and progesterone levels with risk of hormone receptor-positive breast cancers
Source: Br J Cancer. 2021 Jan 26;124(4):842–54. doi: 10.1038/s41416-020-01185-w (PMC7884683; doi:10.1038/s41416-020-01185-w)
Supplement: Supplementary file 1 — Supplemental material [file 41416_2020_1185_MOESM1_ESM.pdf]

Figure S1: QQ plots for single nucleotide polymorphism (SNP) associations with (A) luteal phase urinary oestrone-3-glucuronide and (B) pregnanediol-3-glucuronide levels in 560 premenopausal women. Observed versus expected quantiles are plotted for all autosomal SNPs.

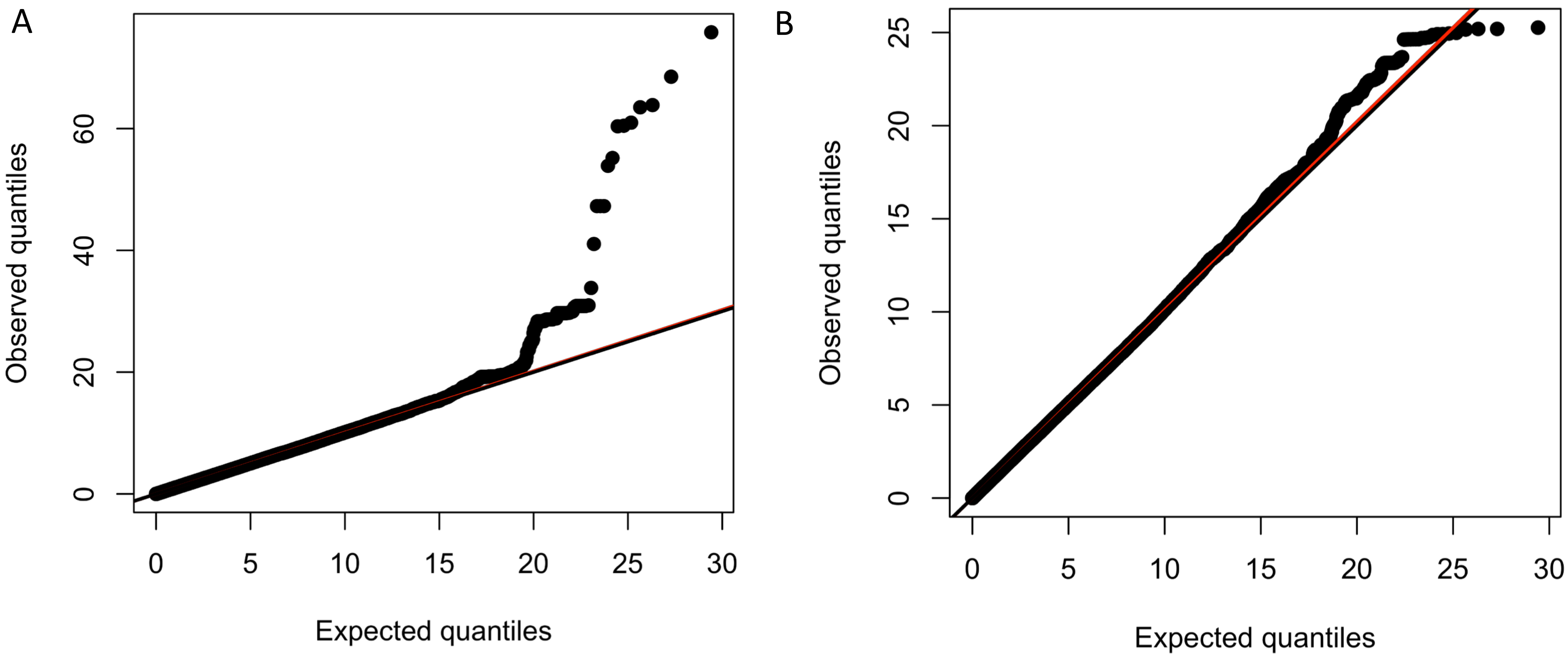

Figure S2: Manhattan plot of single nucleotide polymorphism (SNP) associations with luteal phase pregnanediol-3-glucuronide levels in 560 premenopausal women. $-\log_{10}$ *P*-values for SNP associations are plotted against the genomic coordinates (hg19). The red line indicates the conventionally accepted threshold for genome wide significance (*P*=1 x 10^{-8}).

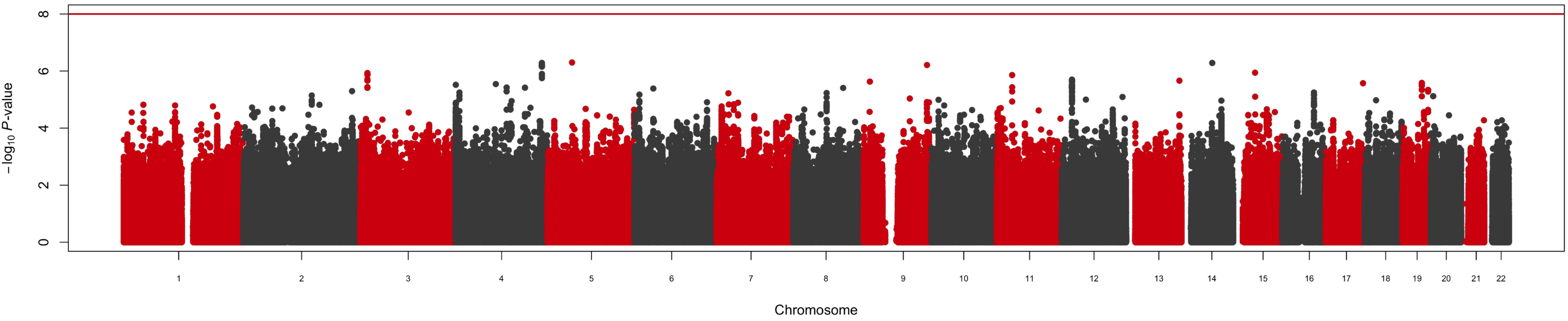

Table S1: rs45446698 genotypes in breast cancer cases and controls from 67 BCAC studies included in the OncoArray collaboration.

Study	Controls AA	Controls AC	Controls CC	Cases AA	Cases AC	Cases CC	MAF[1]	HWE
**EUROPEAN[2]**								
ABCFS	171	16	0	1025	89	3	0.043	1.00
ABCS	173	16	0	321	26	0	0.042	1.00
ABCTB	339	35	1	869	78	0	0.049	0.61
AHS	1041	86	3	475	34	0	0.041	0.43
BBCC	242	11	0	386	17	0	0.022	1.00
BBCS	415	27	0	114	8	0	0.031	1.00
BCEES	779	56	0	728	53	1	0.034	1.00
BCINIS	693	30	1	1244	91	2	0.022	0.30
BREOGAN	663	62	0	1165	98	3	0.043	0.64
BSUCH	160	8	0	237	15	0	0.024	1.00
CBCS	749	67	1	532	35	1	0.042	1.00
CCGP	314	18	0	635	31	1	0.027	1.00
CECILE	150	8	0	255	23	1	0.025	1.00
CGPS	657	58	1	1286	117	5	0.042	1.00
CPSII	2777	241	5	2226	155	3	0.042	1.00
CTS	557	52	1	1091	63	2	0.044	1.00
EPIC	3378	261	1	3222	206	3	0.036	0.09
ESTHER	171	16	0	276	14	1	0.043	1.00
FHRISK	266	22	1	84	16	1	0.042	0.39
GC-HBOC	1468	120	1	3188	215	4	0.038	0.73
GENICA	266	16	1	424	35	0	0.032	0.24
GESBC	168	12	1	277	35	0	0.039	0.23
HABCS	808	57	1	855	52	1	0.034	1.00
HEBCS	150	27	0	259	22	0	0.076	0.60
HMBCS	231	16	1	197	15	0	0.036	0.27
HUBCS	109	11	0	203	8	0	0.046	1.00
KARMA	5539	473	12	2162	179	4	0.041	0.51
KBCP	212	31	2	461	59	2	0.071	0.35
LMBC	1168	94	2	721	62	0	0.039	0.71
MABCS	81	11	0	84	5	0	0.060	1.00
MARIE	271	17	0	478	27	1	0.030	1.00
MBCSG	352	13	1	516	31	2	0.020	0.14
MCBCS	205	16	0	681	66	2	0.036	1.00
MCCS	892	85	0	797	68	5	0.044	0.25
MEC	684	38	1	621	43	4	0.028	0.43
MISS	1398	113	5	548	40	0	0.041	0.10
MMHS	1508	123	3	251	23	1	0.039	0.74
MTLGEBCS	165	5	0	321	19	0	0.015	1.00
NBHS	604	46	0	451	28	2	0.035	1.00
NC-BCFR	144	5	1	683	70	0	0.023	0.07
NCBCS	930	58	1	1908	132	3	0.030	0.60
NHS	1665	135	2	1030	71	0	0.039	1.00
NHS2	1763	132	3	1041	68	0	0.036	0.74
OFBCR	346	29	0	1538	120	3	0.039	1.00
ORIGO	613	45	2	848	71	0	0.037	0.22
PBCS	1895	145	1	1603	130	3	0.036	0.52
PLCO	2364	209	0	1660	140	1	0.041	0.02
PROCAS	1508	133	6	302	27	0	0.044	0.13
RBCS	221	19	0	423	27	0	0.040	1.00
SEARCH	2485	182	4	3742	308	4	0.036	0.58
SISTER	1444	116	0	1420	80	2	0.037	0.27
SMC	650	52	2	1402	102	4	0.040	0.30
SZBCS	163	11	0	327	25	0	0.032	1.00
UCIBCS	243	13	0	400	26	0	0.025	1.00
UKBGS	648	57	0	964	82	1	0.040	0.62
USRT	1569	123	2	1253	90	1	0.037	1.00
**ASIAN**								
ACP	639	3	0	447	1	0	0.002	N/A
CBCS	170	0	0	252	0	0	0.000	N/A
HERPACC	282	0	0	231	0	0	0.000	N/A
HKBCS	454	0	0	477	0	0	0.000	N/A
KOHBRA	665	0	0	1292	0	0	0.000	N/A
MYBRCA	1257	1	0	832	0	0	0.0004	N/A
NC-BCFR	52	0	0	443	1	0	0.000	N/A
NGOBCS	366	0	0	366	0	0	0.000	N/A
SBCGS	935	0	0	815	0	0	0.000	N/A
SEBCS	1107	0	0	1102	0	0	0.000	N/A
SGBCC	704	0	0	711	0	0	0.000	N/A
TWBCS	256	0	0	514	0	0	0.000	N/A
**AFRICAN[2]**								
MEC	711	13	0	631	10	0	0.009	N/A
NBHS	139	4	0	152	0	0	0.014	N/A
NCBCS	587	9	0	1903	26	0	0.008	N/A
SISTER	167	2	0	97	3	0	0.006	N/A
WAABCS	305	2	0	311	1	0	0.003	N/A

[1]MAF (minor allele frequency) is calculated based on controls only.
[2]Excludes 18 European, 1 mixed ethicity and 3 African studies for which there were less than 50 cases or 50 controls.
HWE= *P* -value for test of departure from Hardy-Weinberg equilibrium in controls.

Table S2: Imputed rs45446698 genotypes in breast cancer cases and controls from 45 BCAC studies genotyped in the iCOGS collaboration.

Study	Controls AA	Controls AC	Controls CC	Cases AA	Cases AC	Cases CC	MAF[1]	HWE
**EUROPEAN[2]**								
ABCFS	495	44	2	302	20	0	0.044	0.28
ABCS	1518	97	1	722	47	0	0.031	1.00
BBCC	429	21	0	412	24	0	0.023	1.00
BBCS	1293	93	1	1300	93	3	0.034	1.00
BIGGS	656	55	3	738	51	2	0.043	0.13
BSUCH	904	37	0	682	44	1	0.020	1.00
CECILE	780	55	2	582	39	3	0.035	0.27
CGPS	4144	347	5	2631	212	3	0.040	0.56
CNIO-BCS	801	59	2	802	59	1	0.037	0.31
CPSII	270	20	0	130	7	0	0.034	1.00
ESTHER	287	23	1	169	12	0	0.040	0.39
GENICA	396	25	1	426	23	0	0.032	0.35
HEBCS	970	77	3	1352	135	2	0.040	0.22
HMBCS	90	4	0	490	39	0	0.021	1.00
KARBAC	606	45	2	290	15	0	0.038	0.23
KCONFAB/AOCS	806	81	2	430	28	0	0.048	1.00
LMBC	511	41	0	2438	162	3	0.037	1.00
MARIE	1640	118	2	1076	54	1	0.035	1.00
MBCSG	376	21	0	178	9	0	0.026	1.00
MCBCS	1662	153	3	1213	96	2	0.044	1.00
MCCS	204	22	0	180	17	0	0.049	1.00
MEC	123	6	0	96	8	1	0.023	1.00
MTLGEBCS	271	20	1	169	17	1	0.038	0.34
NBCS	254	21	0	1177	105	2	0.038	1.00
NBHS	74	4	0	83	6	0	0.026	1.00
OBCS	377	24	3	458	33	2	0.037	0.01
OFBCR	326	24	1	463	24	0	0.037	0.38
ORIGO	308	15	0	299	18	0	0.023	1.00
PKARMA	4987	376	4	3949	262	6	0.036	0.32
RBCS	650	35	1	550	37	1	0.027	0.39
SASBAC	1266	93	2	1053	67	0	0.036	0.69
SBCS	787	54	4	707	41	0	0.037	0.02
SEARCH	5712	462	12	8100	566	9	0.039	0.40
SZBCS	277	19	0	308	22	0	0.032	1.00
TNBCC	393	28	0	433	33	3	0.033	1.00
**ASIAN**								
ACP	632	3	0	414	1	0	0.002	N/A
HERPACC	1369	3	0	557	0	0	0.001	N/A
LAABC	988	0	0	804	2	0	0.000	N/A
MYBRCA	597	8	0	581	1	0	0.007	N/A
SBCGS	892	0	0	825	2	0	0.000	N/A
SEBCS	1129	0	0	1018	1	0	0.000	N/A
SGBCC	502	0	0	64	0	0	0.000	N/A
TWBCS	236	0	0	776	0	0	0.000	N/A
**AFRICAN**								
NBHS	241	7	0	258	3	0	0.014	N/A
SCCS	661	9	0	662	10	0	0.007	N/A

[1]MAF (minor allele frequency) is calculated based on controls only.

[2]Excludes 9 European studies with less than 50 cases or 50 controls after excluding samples that were genotyped in iCOGS and OncoArray.

HWE = *P* -value for test of departure from Hardy-Weinberg equilibrium in controls.

Table S3: Details of 14 SNPs and indels mapping to the CYP3A locus and which were associated with E1G levels in 560 premenopausal women (P <1x10^{-8})[1].

rsid	position[2]	MAF	imputed or genotyped	info (imputed SNPs)	effect size (%)	95% CI	*P* value
rs78653058:98838805:G:T	98,838,805	0.021	imputed	0.955	-48.36	-37.03 to -57.66	1.50E-10
rs35188655:98863749:C:CA	98,863,749	0.025	imputed	0.965	-49.09	-39.30 to -57.30	2.10E-13
rs139380031:98911827:C:A	98,911,827	0.025	imputed	0.966	-49.08	-39.42 to -57.19	1.10E-13
7:99025328:C:T	99,025,328	0.033	imputed	0.991	-48.51	-39.69 to -56.04	1.30E-15
rs148982377:99075038:T:C	99,075,038	0.035	genotyped		-47.54	-38.30 to -55.03	1.60E-15
rs117978821:99107775:T:C	99,107,775	0.029	imputed	0.991	-49.36	-40.16 to -57.15	7.80E-15
rs34670419	99,130,834	0.035	genotyped		-46.89	-38.02 to -54.49	5.80E-15
rs10278040	99,141,373	0.055	genotyped		-32.10	-22.79 to -40.28	6.00E-09
rs111390579:99173193:TAC:TACACAC	99,173,193	0.028	imputed	0.888	-52.04	-42.57 to -59.94	7.50E-15
rs118168183:99291144:G:A	99,291,144	0.030	imputed	0.994	-51.38	-42.63 to -58.79	1.30E-16
**rs45446698[3]**	99,332,948	0.038	genotyped		-49.17	-41.11 to -56.12	3.10E-18
**rs45467892:99332997:T:A**	99,332,997	0.022	imputed	0.832	-53.90	-42.79 to -62.86	6.20E-12
**rs45575938:99332998:A:G**	99,332,998	0.022	imputed	0.832	-53.90	-42.79 to -62.86	6.20E-12
**rs45494802:99333000:A:T**	99,333,000	0.022	imputed	0.832	-53.90	-42.79 to -62.86	6.20E-12

[1]For this analysis we used a threshold of 1 x 10^{-8} rather than the conventionally accepted threshold for genome-wide significance of 5 x 10^{-8}.
By doing so we have excluded a cluster of 12 additional highly correlated CYP3A locus SNPs with 1 x 10-8 < P < 5 x 10-8.

[2]positions are based on hg19.

[3]adjusting for age at urine collection (<35, 35-40, ≥40 years), age at menarche (<12, 12, 13, 14, >14 years), BMI (<18.5, 18.5 - <20.0, 20.0 - <25.0, 25.0 - <30.0, ≥ 30.0 kg/m2) , parity (0, 1, 2, ≥3 live births) the result was -47.6%, 95% CI -37.1 to -56.4, P = 3.7 x 10-12.

MAF=minor allele frequency.

4 SNPs that comprise part of the *CYP3A7*1C* allele are in bold.

Sequencing of the *CYP3A7*1C* allele in 31 women (9 common homozygotes and 22 carriers) confirmed the rs45446698 genotype for all 31.

Of the 22 carriers, 8 were heterozygous for rs45446698 (by GWAS genotyping) but not for the other 3 SNPs (by imputation).

Sequencing demonstrated that these 8 women were also heterozygous for the three imputed SNPs; all four are in complete linkage but imputation undercalls rs45467892, rs45575938, rs45494802 resulting in a lower estimate of the MAF and a less significant *P* value compared to rs45446698.

Table S4: Association of rs45446698 genotype with breast cancer risk among women of European ancestry stratified by HER2 status, grade and stage.

	iCOGS					OncoArray					Combined				
	**Cases**	**Controls**	**OR**	**95% CI**	***P*** $_1$	**Cases**	**Controls**	**OR**	**95% CI**	***P*** $_1$	**Cases**	**Controls**	**OR**	**95% CI**	***P*** $_1$
Her2+	1,483	15,909	0.89	0.71 - 1.11	0.3	2,162	18,598	0.98	0.82 - 1.18	0.85	3,645	34,507	0.95	0.82 - 1.09	0.43
Her2-	8,855	15,909	0.89	0.79 - 1.00	0.04	10,182	18,598	1.01	0.92 - 1.12	0.77	19,037	34,507	0.95	0.88 - 1.02	0.18
NK	8,661					10,928									
**Total**	18,999	15,909		P_{int} = 0.72		23,272	18,598		P_{int} = 0.87					P_{int} = 0.74	
Grade 1	3,084	15,909	0.87	0.75 - 1.02	0.09	3,764	18,598	0.92	0.80 - 1.06	0.24	6,848	34,507	0.90	0.81 - 1.00	0.04
Grade 2	7,587	15,909	0.96	0.86 - 1.07	0.43	8,389	18,598	0.97	0.88 - 1.08	0.60	15,976	34,507	0.97	0.90 - 1.04	0.38
Grade 3	5,240	15,909	0.85	0.74 - 0.97	0.01	5,505	18,598	1.00	0.89 - 1.13	0.98	10,745	34,507	0.92	0.84 - 1.00	0.06
NK	3,088					5,614									
**Total**	18,999	15,909		P_{int} = 0.11		23,272	18,598		P_{int} = 0.33					P_{int} = 0.90	
Stage I	7,732	15,909	0.91	0.81 - 1.01	0.09	8,848	18,598	0.96	0.87 - 1.07	0.46	16,580	34,507	0.94	0.87 - 1.01	0.08
Stage II	7,185	15,909	0.90	0.80 - 1.01	0.07	6,599	18,598	0.96	0.86 - 1.08	0.50	13,784	34,507	0.92	0.85 - 1.00	0.06
Stage III	1,594	15,909	0.91	0.73 - 1.13	0.39	1,634	18,598	1.21	1.01 - 1.46	0.05	3,228	34,507	1.06	0.92 - 1.22	0.4
Stage IV	313	15,909	N/A	N/A	N/A	379	18,598	N/A	N/A	N/A	692	34,507	N/A	N/A	N/A
NK	2,175					5,812									
**Total**	18,999	15,909		P_{int} = 0.94		23,272	18,598		P_{int} = 0.09		42,271	34,507		P_{int} = 0.33	

P_1 = test of H_0 no association between rs45446698 and breast cancer risk.

P_{int} = test of H_0 no difference between stratum specific estimates.

NK = not known.

Studies with less than 50 cases in any stratum were excluded from the stratified analyses leaving 10 studies for analysis in iCOGS data and 13 studies for analysis in OncoArray data.

Table S5: Association of rs45446698 genotype with ER+/PR+ breast cancer risk among women of European ancestry in studies with additional covariate data.

	iCOGS					OncoArray					Combined				
	Cases	Controls	OR	95% CI	P_1	Cases	Controls	OR	95% CI	P_1	Cases	Controls	OR	95% CI	P_1
**ER+/PR+ Risk**															
All women															
Unadjusted	8,131	19,715	0.79	0.71-0.89	4.42E-05	17,075	40,866	0.93	0.86-1.00	0.05	25,206	60,581	0.88	0.83-0.94	9.53E-05
Adjusted[1]	6,950	16,850	0.78	0.69-0.88	4.59E-05	14,804	34,636	0.93	0.85-1.01	0.07	21,754	51,486	0.87	0.82-0.94	0.0001
Premenopausal women															
Unadjusted	2,174	6,161	0.90	0.72-1.12	0.36	5,201	13,983	0.96	0.83-1.10	0.53	7,375	20,144	0.94	0.84-1.06	0.31
Adjusted[1]	1,757	5,140	0.88	0.69-1.13	0.32	4,419	10,817	0.98	0.84-1.15	0.81	6,176	15,957	0.95	0.83-1.09	0.46
Postmenopausal women															
Unadjusted	5,957	13,554	0.77	0.68-0.88	7.89E-05	11,874	26,883	0.91	0.83-1.00	0.06	17,831	40,437	0.86	0.80-0.93	0.0001
Adjusted[1]	5,193	11,710	0.76	0.66-0.88	0.0001	10,385	23,819	0.9	0.81-1.00	0.04	15,578	35,529	0.85	0.78-0.92	9.06E-05

P_1 = test of H_0 no association between rs45446698 and ER+/PR+breast cancer risk.

All analyses are adjusted for study and ten principal components.

[1]Adjusted for reference age, age at menarche (<12, 12, 13, 14, >14 years), BMI (<18.5, 18.5 - <20.0, 20.0 - <25.0, 25.0 - <30.0, ≥ 30.0 kg/m2), parity (0, 1, 2, ≥3 live births).

Studies with less than 50 cases in any stratum were excluded from the stratified analyses leaving 13 studies for analysis in iCOGS data and 27 studies for analysis in OncoArray data.

Testing for heterogeneity in estimates between pre- and postmenopausal women, P_{int}=0.28.

Table S6: Association of rs45446698 with breast cancer specific survival in breast cancer cases of European Ancestry stratified by hormone receptor status, Her2 status, grade and stage.

Group	iCOGS					OncoArray					Combined			
	HR	95% CI	P_1	Cases	Events	HR	95% CI	P_1	Cases	Events	HR	95% CI	P_1	P_{het}
ER-	0.93	0.691-1.255	0.64	5,227	646	1.07	0.861-1.34	0.53	9,127	1,068	1.02	0.853-1.218	0.83	0.45
ER+	0.90	0.729-1.106	0.30	22,530	1,481	1.07	0.924-1.245	0.36	41,273	2,268	1.01	0.892-1.138	0.91	0.17
PR-	0.90	0.674-1.198	0.46	7,155	733	1.00	0.831-1.209	0.98	14,176	1,452	0.97	0.829-1.134	0.70	0.53
PR+	0.99	0.753-1.304	0.95	15,842	844	1.10	0.919-1.321	0.30	31,063	1,563	1.07	0.915-1.242	0.41	0.53
HER2+	0.93	0.712-1.202	0.55	13,453	973	1.10	0.914-1.314	0.33	28,052	1,543	1.04	0.891-1.203	0.65	0.29
HER2-	1.22	0.77-1.927	0.41	2,349	242	0.94	0.68-1.308	0.72	5,748	544	1.02	0.784-1.337	0.86	0.38
Grade 1	0.94	0.482-1.827	0.85	5,353	134	1.39	0.859-2.246	0.20	9,367	166	1.20	0.806-1.794	0.37	0.35
Grade 2	0.92	0.702-1.215	0.56	12,666	838	0.89	0.715-1.113	0.30	22,744	1,231	0.90	0.763-1.071	0.24	0.85
Grade 3	1.07	0.845-1.351	0.59	8,152	1,020	1.09	0.905-1.305	0.38	14,694	1,600	1.08	0.933-1.249	0.30	0.91
Stage I	1.05	0.712-1.546	0.81	10,491	369	0.88	0.576-1.328	0.52	14,186	343	0.96	0.725-1.276	0.79	0.53
Stage II	0.88	0.698-1.117	0.29	10,294	1,164	1.09	0.867-1.365	0.47	13,116	1,003	0.98	0.833-1.154	0.82	0.21
Stage III	1.15	0.78-1.68	0.49	1,898	349	0.84	0.61-1.17	0.30	2,942	524	0.95	0.75-1.22	0.71	0.23
Stage IV	0.85	0.45-1.61	0.61	386	156	1.05	0.71-1.56	0.80	577	312	0.99	0.71-1.38	0.96	0.57

ER=estrogen receptor, PR=progesterone receptor.

P_{het} = test of H_0 no difference across genotyping platforms.

38 studies from iCOGS and 63 studies from OnocoArray provided follow up data for analysis of breast cancer specific survival.

Results were censored at 10 years after diagnosis.

Table S7: Ethical review boards providing approval for each BCAC study.

Study	Acronym	Country	Approval Committee(s)
Australian Breast Cancer Family Study	ABCFS	Australia	The University of Melbourne Health Sciences Human Ethics Sub-Committee (HESC)
Amsterdam Breast Cancer Study	ABCS	Netherlands	Leiden University Medical Center (LUMC) Commissie Medische Ethiek; Protocol Toetsingscommissie van Het Nederlands Kanker Instituut-Antoni van Leeuwenhoek Ziekenhuis
Australian Breast Cancer Tissue Bank	ABCTB	Australia	Sydney Local Health District (RPA Zone) Research Ethics and Governance Office
Agricultural Health Study	AHS	USA	National Institute of Health (NIH) Population Sciences IRB
Bavarian Breast Cancer Cases and Controls	BBCC	Germany	Friedrich-Alexander-Universitat Erlangen-Nurnberg Medizinische Fakultat Ethik-Commission
British Breast Cancer Study	BBCS	UK	South East Multi-Centre Research Ethics Committee
Breast Cancer Environment and Employment Study	BCEES	Australia	The University of Western Australia Human Ethics Research Committee
Breast Cancer In Northern Israel Study	BCINIS	Israel	Carmel Medical Center
Breast Cancer In Galway Genetic Study	BIGGS	Ireland	Galway University College Hospital Clinical Research Ethical Committee
Breast Oncology Galicia Network	BREOGAN	Spain	Comité Autonómico de Ética de la Investigación de Galicia
Breast Cancer Study of the University Clinic Heidelberg	BSUCH	Germany	Ethikkommission Medizinische Fakultat Heidelberg, University of Heidelberg
Canadian Breast Cancer Study	CBCS	Canada	University of British Columbia - BC Cancer Research Ethics Board; Queen's University Health Sciences and Affiliated Teaching Hospitals Research Human Ethics Board (HSREB)
Crete Cancer Genetics Program	CCGP	Greece	Epistimoniko Symvoulio (Scientific Council of the University General hospital of Heraklion)

CECILE Breast Cancer Study	CECILE	France	Comité Consultatif de Protection des Personnes dans la Recherche Biomédicale de Bicêtre (Le Kremlin-Bicêtre FR-94270)
Copenhagen General Population Study	CGPS	Denmark	Kobenhavns Amt den Videnskabsetiske Komite (Scientific ethical committee, Copenhagen County)
Spanish National Cancer Centre Breast Cancer Study	CNIO-BCS	Spain	Comité de ética de la Investigacion y de Bienestrar Animal del Insituto de Salud Carlos III
Cancer Prevention Study-II Nutrition Cohort	CPSII	USA	Emory University Institutional Review Board
California Teachers Study	CTS	USA	UC Irvine: Office of Research Institutional Review Board
European Prospective Investigation Into Cancer and Nutrition	EPIC	Various within EU	Institutional Review Board of the International Agency for Research on Cancer, Lyon, France; Ethics Commission of the Faculty of Medicine of the University of Heidelberg
ESTHER Breast Cancer Study	ESTHER	Germany	Ruprecht-Karls-Universitat Medizinische Fakultat Heidelberg Ethikkommission
Family History Risk Study	FHRISK	UK	NRES Committee North West - Greater Manchester Central
German Consortium for Hereditary Breast & Ovarian Cancer	GC-HBOC	Germany	Ethik-Kommission der Medizinischen Fakultat der Universitat zu Koln
Gene Environment Interaction and Breast Cancer in Germany	GENICA	Germany	Ethikkommission Rheinische Friedrich-Wilhels-Universität Bonn
Genetic Epidemiology Study of Breast Cancer by Age 50	GESBC	Germany	Medizinische Fakultat Heidelberg Ethikkommission
Hannover Breast Cancer Study	HABCS	Germany	Medizinische Hochschule Hannover Ethik-Kommission
Helsinki Breast Cancer Study	HEBCS	Finland	Helsingin ja uudenmaan sairaanhoitopiiri (Helsinki University Hospital Ethics Committee)
Hannover-Minsk Breast Cancer Study	HMBCS	Belarus	Medizinische Hochschule Hannover Ethik-Kommission
Hannover-Ufa Breast Cancer Study	HUBCS	Russia	Ethical Committee of Institute of Biochemistry and Genetics, Ufa Scientific Center of Russian Academy of Sciences
Karolinska Breast Cancer Study	KARBAC	Sweden	Regionala Etikprovningsnamnden i Stockholm (Regional Ethical Review Board in Stockholm)

Karolinska Mammography Project for Risk Prediction of Breast Cancer - Cohort Study	KARMA	Sweden	Regionala Etikprovningsnamnden i Stockholm (Regional Ethical Review Board in Stockholm)
Kuopio Breast Cancer Project	KBCP	Finland	Pohjois-Savon Sairraanhoitopiirin Kuntayhtyma Tutkimuseettinen Toimikunta
Kathleen Cuningham Foundation Consortium for Familial Breast Cancer/Australian Ovarian Cancer Study	kConFab/AOCS	Australia	kConFab: Peter MacCallum Cancer Centre Ethics Committee; AOCS: Peter MacCallum Cancer Centre Ethics Committee
Leuven Multidisciplinary Breast Centre	LMBC	Belgium	Commissie Medische Ethiek van de Universitaire Ziekenhuizen Kuleuven
Macedonian Breast Cancer Study	MABCS	Republic of North Macedonia	Ethic Subcommittee of Medicine, Pharmacy, Veterinary Medicine and Dentistry, Macedonia Academy of Sciences and Arts
Mammary Carcinoma Risk Factor Investigation	MARIE	Germany	Medizinische Fakultat Heidelberg Ethikkommission; Ethik-Kommission der Arztekammer Hamburg
Milan Breast Cancer Study Group	MBCSG	Italy	Comitato Etico Indipendente della Fondazione IRCCS "Istituto Nazionale dei Tumori"
Mayo Clinic Breast Cancer Study	MCBCS	USA	Mayo Clinic IRB
Melbourne Collaborative Cohort Study	MCCS	Australia	The Cancer Council Victoria Human Research Ethics Committee
Multi-ethnic Cohort	MEC	USA	University of Southern California Health Sciences Campus IRB
Melanoma Inquiry of Southern Sweden	MISS	Sweden	Regional Ethical Board in South Sweden
Mayo Mammography Health Study	MMHS	USA	Mayo Clinic IRB
Montreal Gene-Environment Breast Cancer Study	MTLGEBCS	Canada	McGill University IRB
Norwegian Breast Cancer Study	NBCS	Norway	Regionale Komitere for Medisinsk og Helsefaglig Forskningsetikk
Nashville Breast Health Study	NBHS	USA	Vanderbilt University Medical Center IRB
Northern California Breast Cancer Family Registry	NC-BCFR	USA	Stanford University IRB

North Carolina Breast Cancer Study	NCBCS	USA	Office of Human Research Ethics, the University of North Carolina, Chapel Hill
Nurses Health Study	NHS	USA	Partners Human Research, Partners Healthcare System (PHS) IRB
Nurses Health Study 2	NHS2	USA	Partners Human Research, Partners Healthcare System (PHS) IRB
Oulu Breast Cancer Study	OBCS	Finland	Pohjois-Pohjenmaan Sairaanhoitopiirin Kuntayhtymä: Alueellinen Eettinen Toimikunta; Terveyden Ja Hyvinvoinnin Laitos (Finnish National Institute For Health And Welfare)
Ontario Familial Breast Cancer Registry	OFBCR	Canada	Mount Sinai Hospital Research Ethics Board
Leiden University Medical Centre Breast Cancer Study	ORIGO	Netherlands	Medical Ethical Committee and Board of Directors of the Leiden University Medical Center (LUMC)
NCI Polish Breast Cancer Study	PBCS	Poland	National Cancer Institute Special Studies Institutional Review Board (NCI-SSIRB)
Karolinska Mammography Project for Risk Prediction of Breast Cancer - Case-Control Study	pKARMA	Sweden	Regionala Etikprovningsnamnden i Stockholm (Regional Ethical Review Board in Stockholm)
The Prostate, Lung, Colorectal and Ovarian (PLCO) Cancer Screening Trial	PLCO	USA	National Cancer Institute Special Studies Institutional Review Board (NCI-SSIRB)
Predicting the Risk Of Cancer At Screening Study	PROCAS	UK	NRES Committee North West - Greater Manchester Central
Rotterdam Breast Cancer Study	RBCS	Netherlands	Medische Ethische Toetsings Commissie Erasmus Medisch Centrum
Singapore and Sweden Breast Cancer Study	SASBAC	Sweden	Regionala Etikprovningsnamnden i Stockholm (Regional Ethical Review Board in Stockholm)
Sheffield Breast Cancer Study	SBCS	UK	Yorkshire & The Humber - Sheffield Research Ethics Committee
Study of Epidemiology and Risk factors in Cancer Heredity	SEARCH	UK	Multi Centre Research Ethics Committee (MREC)
The Sister Study	SISTER	USA	Institutional Review Board of the National Institute of Environmental Health Sciences; NIH and the Copernicus Group Independent Review Board

Swedish Mammography Cohort	SMC	Sweden	Regionala Etikprovningsnamnden i Stockholm (Regional Ethical Review Board in Stockholm)
IHCC-Szczecin Breast Cancer Study	SZBCS	Poland	Komisji Bioetycznej Pomorskiej Akademii Medycznej
Triple Negative Breast Cancer Consortium Study	TNBCC	Various	Mayo Clinic IRB
UCI Breast Cancer Study	UCIBCS	USA	UC Irvine: Office of Research Institutional Review Board
UK Breakthrough Generations Study	UKBGS	UK	London- South East Research Ethics Committee
US Radiologic Technologists Study	USRT	USA	National Cancer Institute Special Studies Institutional Review Board (NCI-SSIRB)
